# NAD^+^ Enhanced Mesenchymal Stromal Cells Effect on Muscle Atrophy by Improving SIRT1‐Mediated Mitochondrial Function via NAMPT

**DOI:** 10.1002/jcsm.70142

**Published:** 2025-12-12

**Authors:** Jia Song, Yuting Sun, Nan Zang, Xue Liu, Jiamu Chen, Kewei Wang, Longqing Xia, Jun Chen, Ruxing Zhao, Fuqiang Liu, Xinguo Hou, Li Chen, Jun Cheng, Wenjian Zhang

**Affiliations:** ^1^ Department of Endocrinology and Metabolism Qilu Hospital of Shandong University Jinan Shandong China; ^2^ Shandong Provincial Key Laboratory of Spatiotemporal Regulation and Precision Intervention in Endocrine and Metabolic Diseases; Shandong Provincial Engineering Research Center for Advanced Technologies in Prevention and Treatment of Chromic Metabolic Diseases; Institute of Endocrine and Metabolic Diseases of Shandong University Jinan Shandong China; ^3^ The First Clinical Medical College, Cheeloo College of Medicine Shandong University Jinan Shandong China; ^4^ Department of Clinical Laboratory, Shandong Engineering & Technology Research Center for Tumor Marker Detection The Second Qilu Hospital of Shandong University Jinan Shandong China

**Keywords:** mitochondrial function, muscle atrophy, NAD^+^‐MSCs, NAMPT, SIRT1/PGC‐1α

## Abstract

**Background:**

Sarcopenia contributes to all‐cause mortality in the elderly; however, there is no specific treatment. Mesenchymal stromal cells (MSCs) ameliorate age‐related muscle loss and dysfunction and are potential therapeutic candidates for sarcopenia. However, their activity is easily affected by the surrounding environment and they are prone to replicative senescence during in vitro culture. Therefore, a drug that delays aging and enhances its function is required. Here, we investigated whether nicotinamide adenine dinucleotide (NAD^+^) pretreatment enhances the therapeutic efficacy of MSCs on skeletal muscle atrophy and its underlying mechanism in a D‐galactose (D‐gal)–induced mouse model.

**Methods:**

The administration of D‐gal to mice induces a range of age‐associated characteristics and is commonly used in research on age‐related muscle atrophy. Therefore, in this study, C57BL/6 J mice and C2C12‐differentiated myotubes exposed to D‐gal were used to explore the effects of MSCs/NAD^+^‐MSCs on muscle atrophy. MSCs/NAD^+^‐MSCs were injected into the skeletal muscles of the hind limbs every 7 days for six cycles. Treadmill running and grip strength tests were used to evaluate muscle strength. Muscle weight and fibre cross‐sectional area (CSA) were used to measure muscle mass. Multiomics analysis of quadriceps and NAD^+^‐pretreated MSCs (NAD^+^‐MSCs), Western blotting of muscle atrophy signalling, including Atrogin 1 and MuRF1, the mitochondrial complex, fatty acid oxidation indicators and Seahorse analysis were performed to explore the underlying mechanisms.

**Results:**

MSCs increased grip strength (*p* = 0.0005), running endurance (*p* = 0.0006) and muscle mass (*p* = 0.0165 for tibialis anterior [TA] muscle, *p* = 0.0049 for soleus [SO] muscle) in D‐gal–treated mice, with elevated muscle fibre CSA (*p* < 0.0001) and reduced Atrogin 1 (*p* = 0.0242) and MuRF1 expression (*p* = 0.0009). NAD^+^ pretreatment increased the effect of MSCs on muscle atrophy (*p* = 0.0009 for grip strength, *p* = 0.0169 for running endurance, *p* = 0.0506 for TA muscle weight, *p* = 0.0238 for SO muscle weight, *p* = 0.0014 for muscle fibre CSA, *p* = 0.0005 for Atrogin 1 expression and *p* = 0.0223 for MuRF1 expression). MSCs/NAD^+^‐MSCs activated the SIRT1/PGC‐1α signalling, enhanced mitochondrial function and fatty acid oxidation in D‐gal–induced mice and C2C12 myotubes. SIRT1 knockdown weakened the beneficial effects of MSCs/NAD^+^‐MSCs on muscle atrophy. RNA‐seq of MSCs**/**NAD^+^‐MSCs and proteomic analysis of their supernatants revealed that NAD^+^ enhanced the therapeutic effect of MSCs by promoting NAMPT secretion.

**Conclusions:**

NAD^+^ enhances the therapeutic effect of MSCs on D‐gal–induced muscle atrophy by promoting NAMPT secretion, which acts on the SIRT1 signaling pathway, and improves mitochondrial function and fatty acid oxidation in skeletal muscles. This study provides new insights and a theoretical basis for clinical treatment of sarcopenia.

## 1 Introduction

Sarcopenia is a syndrome associated with aging and characterised by reduced muscle mass, decreased strength and/or physical dysfunction. The prevalence of sarcopenia in people over 60 years of age in China reaches up to 14.7% [1]. Sarcopenia leads to impaired balance and endurance and increases the risk of frailty, falls, osteoporosis and fractures [2, 3], which contribute to all‐cause mortality in the elderly [4]. However, there are no specific treatments for it [5]. Therefore, it is important to explore the underlying mechanisms and effective strategies for treating sarcopenia. D‐galactose (D‐gal)–induced model is a systemic and homogeneous aging model with the acceleration of senescence [Data S1]. The administration of D‐gal to animals induces a range of aging‐associated characteristics, including shortened lifespan, increased oxidative stress, mitochondrial DNA mutation and mitochondrial dysfunction, which may be correlated with skeletal muscle atrophy in aging. This model is commonly used in research on senile diseases, such as aging‐associated muscle atrophy and anti‐aging measures for sarcopenia [6, Data S2 and S3].

Mitochondria are the main sites of fatty acid oxidation, a crucial process in lipid catabolism and energy production, and are critical for maintaining normal skeletal muscle function [7]. In the aging state, type I fibres dependent on the oxidative phosphorylation of mitochondria remain unchanged, whereas the number and size of type II fibres dependent on glycolysis in skeletal muscle gradually decrease. The skeletal muscle changes from mainly dependent on glycolytic to oxidative metabolism during aging, contributing to an increased demand for functional mitochondria [8, 9]. Meanwhile, impaired mitochondrial function and abnormal mitochondrial accumulation during aging further interfere with fatty acid β‐oxidation, leading to lipid accumulation in muscle and affecting muscle regeneration [10]. The skeletal muscles of patients with sarcopenia undergo substantial metabolic changes, and differential genes and metabolites are enriched in fatty acid metabolism and the tricarboxylic acid cycle [11]. Reducing lipid accumulation in the muscles can effectively alleviate muscle atrophy [12]. As a nicotinamide adenine dinucleotide (NAD^+^)–dependent histone deacetylase, sirtuin 1 (SIRT1) plays an important role in mitochondrial function and aging, and its activation can effectively reduce sarcopenia [13]. SIRT1 can promote mitochondrial biosynthesis through peroxisome proliferator–activated receptor gamma coactivator‐1α (PGC‐1α) and play a key role in peroxisome proliferator–activated receptor alpha (PPAR‐α)–mediated fatty acid oxidation, linking energy metabolism and aging in skeletal muscle [14].

Mesenchymal stromal cells (MSCs) can ameliorate age‐related muscle loss and dysfunction and have become potential therapeutic candidates for sarcopenia [6, 15, Data S4]. MSC intervention is effective in many diseases through the modulation of mitochondrial function, including diabetic endothelial dysfunction and myocardial infarction, in which SIRT1 and other factors related to mitochondrial function play important roles [16, 17]. Our previous study showed that MSC‐derived exosomes alleviate diabetes‐induced muscle atrophy by improving SIRT1‐mediated mitochondrial function [18]. However, MSC activity is not only easily affected by the surrounding environment but is also prone to replicative senescence during in vitro culture, which limits their clinical application [19]. NAD^+^ is a crucial coenzyme in redox reactions and plays a vital role in various biological processes, including metabolism and aging [20]. NAD^+^ plays a positive role in delaying MSC senescence, promoting osteogenic differentiation of MSCs and enhancing their anti‐inflammatory functions [21, 22, Data S5]. However, further investigation is warranted to determine whether NAD^+^ pretreatment can enhance the therapeutic efficacy of MSCs and elucidate the underlying mechanisms in sarcopenia.

Therefore, in this study, we first established a D‐gal–induced mouse model to assess the protective effects of MSCs/NAD^+^‐MSCs derived from umbilical Wharton's jelly against muscle atrophy. Next, in combination with the results of multi‐omics sequencing analysis, we conducted in vitro studies using C2C12‐differentiated myotubes to further explore the specific underlying mechanisms. Our findings may provide new insights and a theoretical basis for the clinical treatment of muscle atrophy.

## 2 Materials and Methods

### 2.1 Human MSC Isolation, Characterisation and RNAi

MSCs were harvested from the fresh umbilical cords of healthy newborns with informed parental consent, as previously reported [23]. MSCs were cultured in α‐MEM medium (Gibco, NY, USA) supplemented with 10% foetal bovine serum (FBS; Gibco), along with 100 U/mL penicillin and 100 μg/mL streptomycin (Gibco). The third to fifth passages of the cells were used for flow cytometry, induction of differentiation, administration and co‐culture using the Transwell system. NAD^+^‐MSCs were obtained by NAD^+^ stimulation (150 μmol/L) of MSCs for 48 h.

To explore the underlying mechanism of NAD^+^ action, MSCs were transfected with small interfering RNA (siRNA) targeting nicotinamide phosphoribosyltransferase (NAMPT) prior to NAD^+^ exposure using Lipofectamine 2000 transfection reagent (Invitrogen, CA, USA) following the manufacturer's instructions. MSCs were seeded in six‐well plates and then shifted to Opti‐MEM I–reduced serum medium (Gibco) containing NAMPT siRNA (125 nM) for 6 h. Next, the α‐MEM medium was introduced and MSCs were subjected to NAD^+^ treatment for 48 h. The siRNA oligonucleotides were synthesised by GenePharma Co. Ltd. (Shanghai, China). The sequences for the negative control (NC) siRNA were outlined as follows: sense 5′‐UUCUCCGAACGUGUCACGUTT‐3′ and antisense 5′‐ACGUGACACGUUCGGAGAATT‐3′. The sequences for NAMPT siRNA were as follows: sense 5′‐GCAGAACACAGUACCAUAATT‐3′ and antisense 5′‐UUAUGGUACUGUGUUCUGCTT‐3′.

### 2.2 Cell Culture and RNAi

Human embryonic lung fibroblasts (HELFs) were acquired from the China Cell Culture Center (Shanghai, China) and maintained in high‐glucose Dulbecco's modified eagle's medium (DMEM, Gibco), enriched with 10% FBS, 100 U/mL penicillin and 100 μg/mL streptomycin at 37°C in a 5% CO_2_ incubator. Mouse C2C12 myoblasts were purchased from China Infrastructure of Cell Line Resource (Beijing, China) and cultured in high‐glucose DMEM supplemented with 10% FBS and antibiotics. Upon reaching 80%–90% confluence, a differentiation medium comprising DMEM and 2% heat‐inactivated horse serum was introduced for 4 days. Fully differentiated myotubes were subsequently stimulated with D‐gal (20 mg/mL; MCE, Shanghai, China) for 48 h, followed by treatment with MSCs, HELFs and NAD^+^‐MSCs via a Transwell system (Corning, NY, USA) for 24 h.

To validate the role of SIRT1 in MSCs, C2C12 myoblasts were transfected with short hairpin RNA (shRNA, GenePharma Co. Ltd.) using a lentivirus (LV) according to the manufacturer's protocols. The transfected cells were fully differentiated for 4 days and exposed to MSCs/NAD^+^‐MSCs for 24 h. The shRNA oligonucleotides were synthesised by GenePharma Co. Ltd. The sequences for NC shRNA were 5′‐TTCTCCGAACGTGTCACGT‐3′, while the sequences for SIRT1 shRNA were 5′‐TTAACAACCTCTTGATCCC‐3′.

### 2.3 Animal Procedure

Six‐week‐old male C57BL/6 J mice were obtained from Jiangsu Huachuang Sino PharmaTech Co. Ltd. (Suzhou, China) and maintained on a standard diet. The mice were housed in an environment with a controlled 12 h light/dark cycle at 22°C–25°C with 55% ± 5% humidity. Following a 1‐week period of accommodative feeding, mice received daily subcutaneous injections of D‐gal at a dose of 100 mg/kg for 12 weeks. MSCs or NAD^+^‐MSCs (1 × 10^6^ cells/mouse) in PBS were injected into the quadriceps (QUAD), tibialis anterior (TA) and gastrocnemius (GAS) muscles of the hind limbs every 7 days for six cycles. The controls received an injection of PBS at the same volume, devoid of MSCs.

### 2.4 In Vivo Muscle Performance Analysis

One week after the last injection of MSCs/NAD^+^‐MSCs, the exhaustive running distance of mice was evaluated on a treadmill (Xinrun Information Technology Co. Ltd., Shanghai, China). Prior to testing, the mice underwent a two‐day acclimation period. Exercise endurance was assessed by measuring the distance they could run until exhaustion was reached. Initially, the mice underwent a 10‐min running session at a speed of 8 m/min. Subsequently, the treadmill's speed was escalated by 0.2 m/min. Exhaustion was determined when the hind limbs of the mice remained on the electric grid for more than 10 consecutive seconds. The grip strength was measured using an electronic dynamometer (Shanghai Xinrun Information Technology Co. Ltd.). The mice were trained to hold onto a horizontal grid linked to the dynamometer using all four limbs and pulled back horizontally with a steady force. The force exerted on the grid each time a mouse lost its grip was recorded. This assessment was conducted three times for each mouse and the obtained measured values underwent an averaging process.

### 2.5 Tissue Processing

Following 1 week of in vivo muscle performance evaluation, the mice were euthanised using CO_2_. Blood samples were collected and plasma was extracted to measure lipid levels. Subsequently, the bilateral muscles, including QUAD, TA, GAS and soleus (SO), were dissected. The TA and SO muscle weights were normalised to the body weight. One of the fresh QUAD muscle samples was used for RNA‐seq, untargeted metabolomics and ATP content assay, while the other was snap‐frozen in liquid nitrogen and stored at −80°C for protein extraction and oil red O staining. TA muscles were preserved in 4% paraformaldehyde for subsequent histology staining, including haematoxylin–eosin (H&E), sirius red and Masson staining.

### 2.6 Histology Staining

The fixed TA muscles were encased in paraffin and sectioned to a thickness of 5 μm at the maximum cross‐section. After deparaffinisation, sections were subjected to standard H&E staining, sirius red staining and Masson staining. The frozen QUAD muscles were sliced to a 7‐μm thickness at the maximum cross‐section and used for oil red O staining following standard procedures. Images were captured using a microscope (BX53; Olympus, Japan) and quantified using Image‐Pro Plus software. Intramuscular lipid content was represented as the ratio of oil red O–positive area to muscle fibre area.

### 2.7 RNA‐Seq and Real‐Time Quantitative PCR Analysis

Total RNA was isolated from QUAD muscles and MSCs using TRIzol reagent (Invitrogen), following the manufacturer's instructions. Subsequent library preparation and sequencing were performed by Beijing Tsingke Biotechnology Co. Ltd. DESeq2 (v1.26.0) was used to perform differential expression analyses. Gene ontology (GO) enrichment analysis of differentially expressed genes (DEGs) was conducted using the GOseq R package, which relies on the Wallenius non‐central hypergeometric distribution. KOBAS software was employed to assess the statistical enrichment of DEGs within KEGG pathways.

Then, 1 μg of RNA was converted into cDNA through reverse transcription using the Prime Script RT Reagent Kit (Cat. No. RR047A; Takara, Japan). Primers were chemically synthesised by Tsingke Biotechnology Co. Ltd. and the sequences of them were as follows: *Mus‐Gapdh*, sense 5′‐AAGGGCTCATGACCACAGTC‐3′ and antisense 5′‐CAGGGATGATGTTCTGGGCA‐3′; *Mus‐p16*, sense 5′‐GCTCAACTACGGTGCAGATTC‐3′ and antisense 5′‐GCACGATGTCTTGATGTCCC‐3′; *Mus‐p21*, sense 5′‐CCTGGTGATGTCCGACCTG‐3′ and antisense 5′‐CCATGAGCGCATCGCAATC‐3′; *Homo‐Gapdh*, sense 5′‐ACAACTTTGGTATCGTGGAAGG‐3′ and antisense 5′ ‐GCCATCACGCCACAGTTTC‐3′; *Homo‐p16*, sense 5′‐GATCCAGGTGGGTAGAAGGTC‐3′ and antisense 5′‐CCCCTGCAAACTTCGTCCT‐3′; *Homo‐p21*, sense 5′‐CGATGGAACTTCGACTTTGTCA‐3′ and antisense 5′‐GCACAAGGGTACAAGACAGTG‐3′; *Homo‐IL‐1β*, sense 5′ ‐ATGATGGCTTATTACAGTGGCAA‐3′ and antisense 5′‐GTCGGAGATTCGTAGCTGGA‐3′ and *Homo‐IL‐6*, sense 5′‐ACTCACCTCTTCAGAACGAATTG‐3′ and antisense 5′‐CCATCTTTGGAAGGTTCAGGTTG‐3′. Real‐time PCR analysis was conducted utilising the SYBR Green PCR kit (Cat. No. RR420A; Takara). Variations in gene expression were evaluated by employing the comparative CT (2^−ΔΔCt^) approach, and the results were quantified through normalisation against *Gapdh*, which served as the reference control.

### 2.8 Untargeted Metabolomics

Untargeted metabolomics of the QUAD muscles was performed at Beijing Tsingke Biotechnology Co. Ltd. The dataset comprising peak numbers, sample identifiers and normalised peak areas was integrated into the SIMCA16.0.2 software (Sartorius Stedim Data Analytics AB, Umea, Sweden) for comprehensive multivariate statistical analysis. A supervised method known as orthogonal projections to latent structure‐discriminant analysis (OPLS‐DA) was used to facilitate the visual assessment of group segregation and identification of significantly altered metabolites. Additionally, the variable importance in the projection (VIP) scores from the primary principal components of the OPLS‐DA model was extracted. Metabolites exhibiting VIP > 1 and *p* < 0.05 (Student's t‐test) were classified as significantly altered metabolites.

### 2.9 Preparation of MSC Supernatant and Proteomics

Upon reaching 80%–90% confluency, the MSCs were subjected to serum‐free medium, followed by a 24‐h incubation period. Subsequently, the supernatant was harvested and concentrated 20‐fold using 3‐kDa molecular weight cutoff ultrafiltration membranes (Millipore), which was subsequently utilised for proteomic analysis.

Proteomics was conducted using the Vanquish Neo UHPLC system for sample separation. Data‐independent acquisition (DIA) was performed using the Vanquish Neo system (Thermo Fisher Scientific) for chromatographic separation. Raw MS data were analysed using DIA‐NN (v1.8.1) with a library‐free method. A spectral library was generated using the uniprotkb_proteome_UP000005640_human_82 493_20240528.fasta database (82 493 sequences), leveraging advanced neural network algorithms for deep learning. To build a spectral library from DIA data, the Match Between Runs feature was applied, which was subsequently utilised for reanalysis. The false discovery rate of the search outcomes was adjusted to < 1% at both the protein and precursor ion levels. Only the remaining valid identifications were employed for subsequent quantitative analysis.

### 2.10 Isolation of Extracelluar Vesicles

Upon reaching 80%–90% confluence, the MSCs/NAD^+^‐MSCs were cultured in serum‐free medium for 24 h, and the cell supernatants were collected. The cell supernatants were centrifuged at 10,000 × *g* for 1 h and filtered through a 0.22‐μm filter to remove cellular debris. Subsequently, the medium was ultracentrifuged at 100,000 × *g* for 70 min at 4°C to obtain extracelluar vesicles (EVs) used for protein extraction.

### 2.11 Western Blotting

QUAD muscle, C2C12 myotubes, MSCs and EVs were subjected to lysis with a radioimmunoprecipitation assay (RIPA) buffer (P0013B; Beyotime, Shanghai, China). The extracted proteins were subsequently separated and transferred onto polyvinylidene difluoride (PVDF) membranes (IPVH00010 0.45 μm; Millipore, MA, USA). Subsequent to blocking with a 5% skim milk solution at room temperature for 1 h, the membranes underwent overnight incubation with specific primary antibodies at 4°C. After incubation with horseradish peroxidase (HRP)–conjugated secondary antibodies for 1 h at room temperature, protein detection was carried out via enhanced chemiluminescence (ECL) analysis. Band quantitation was performed using the ImageJ software, with normalisation to GAPDH as an internal control.

The following primary antibodies were used: Atrogin 1 (1:5000; Cat. No. 67172‐1‐Ig; Proteintech, IL, USA), MuRF1 (1:1000; Cat. No. 55456‐1‐AP; Proteintech), GAPDH (1:5000; Cat. No. AB0037; Abways, Shanghai, China), SIRT1 (1:1000; Cat. No. 13161‐1‐AP; Proteintech), PGC‐1α (1:1000; Cat. No. ab191838; Abcam, MA, USA), PPAR‐α (1:1000; Cat. No. ab126285; Abcam), acyl‐coenzyme A dehydrogenase medium‐chain (ACADM; 1:1000; Cat. No. A1873; ABclonal, Wuhan, China), acyl‐coenzyme A dehydrogenase long chain (ACADL; 1:1000; Cat. No. A1266; ABclonal), NADH dehydrogenase (ubiquinone) 1 beta subcomplex 8 (NDUFB8; 1:1000; Cat. No. 14794‐1‐AP; Proteintech), succinate dehydrogenase complex subunit B (SDHB; 1:5000; Cat. No. 10620‐1‐AP; Proteintech), ubiquinol‐cytochrome c reductase core protein II (UQCRC2; 1:1000; Cat. No. 14742‐1‐AP; Proteintech), cytochrome c oxidase II (MTCO2; 1:1000; Cat. No. 55070‐1‐AP; Proteintech), ATP synthase, H^+^ transport, mitochondrial F1 complex, alpha subunit 1 (ATP5A1; 1:2000; Cat. No. 14676‐1‐AP; Proteintech), heat shock protein 90 (HSP90; 1:1000; Cat. No. A5027; ABclonal) and NAMPT (1:2000; Cat. No. A0256; ABclonal), CD63 (1:5000; Cat. No. 67605‐1‐Ig; Proteintech).

### 2.12 Transmission Electron Microscopy

The TA muscles were dissected and promptly immersed in a fixative solution containing 1% phosphate‐buffered osmium tetroxide and 2.5% glutaraldehyde. Following the embedding process, the muscles were sectioned and stained with uranyl acetate and lead citrate. Subsequently, transmission electron microscope (JEM‐1200EX II, JEOL, Tokyo, Japan) was employed to capture electron photomicrographs illustrating the ultrastructural details of the TA muscle.

### 2.13 ATP Content Assay

Fresh QUAD muscles were used to detect ATP content using the ATP Content Assay Kit (Cat. No. BC0300; Solarbio, Beijing, China) following the manufacturer's instructions. The ATP content of the muscles was normalised to muscle weight.

### 2.14 NAD^+^ Content Assay

MSCs/NAD^+^‐MSCs were used to detect intracellular NAD^+^ content and NAD^+^/NADH ratio using the NAD^+^/NADH Assay Kit with WST‐8 (Cat. No. S0175; Beyotime, Shanghai, China) according to the manufacturer's instructions.

### 2.15 Seahorse Analysis

The assessment of oxygen consumption rate (OCR) was conducted utilising the Mito Stress Test Kit (Cat. No. 103015‐100; Agilent Technologies, CA, USA) following the manufacturer's instructions. C2C12 myoblasts were seeded at a density of 1 × 10^4^ cells/well into an XF96 cell culture microplate and allowed to fully differentiate. Subsequently, the cultured cells were placed in XF assay medium formulated with Seahorse XF DMEM (pH 7.4), containing 1 mM pyruvate, 10 mM glucose and 2 mM pyruvate glutamine, and treated as indicated. The oligomycin, carbonyl cyanide‐4‐(trifluoromethoxy) phenylhydrazone (FCCP) and antimycin A/rotenone concentrations were 1.5, 1.5 and 0.5 μM, respectively. The OCR was evaluated using a Seahorse XF96 Analyser (Agilent Technologies).

### 2.16 Statistical Analysis

All data were expressed in the form of mean ± SEM Differences between groups were analysed using unpaired Student's *t*‐test or one‐way analysis of variance (ANOVA), followed by Tukey's test. These statistical analyses were performed using GraphPad Prism 8 software. *p* < 0.05 was deemed statistically significant.

## 3 Results

### 3.1 NAD^+^ Pretreatment Enhances the Therapeutic Effect of MSCs on D‐Gal–Induced Muscle Atrophy

In this study, we explored the effects of MSCs/NAD^+^‐MSCs on D‐gal–induced skeletal muscle atrophy (Figure S1A). Flow cytometric analysis revealed that the MSCs exhibited positive results for CD105 and CD73 (> 95%) and negative results for CD34 and HLA‐DR (< 2%) (Figure S1B). MSCs have the potential for multi‐directional differentiation in adipogenesis and osteogenesis, as characterised by oil red O and Alizarin red S staining, respectively (Figure S1C,D). NAD^+^ intervention did not change the surface marker characteristics or adipogenic and osteogenic differentiation abilities of MSCs (Figure S1B–D). However, NAD^+^ reduced the mRNA levels of *p16*, *p21*, *IL‐1β* and *IL‐6* in MSCs, which indicated alleviation of MSC aging (Figure S1E).

**FIGURE 1:**
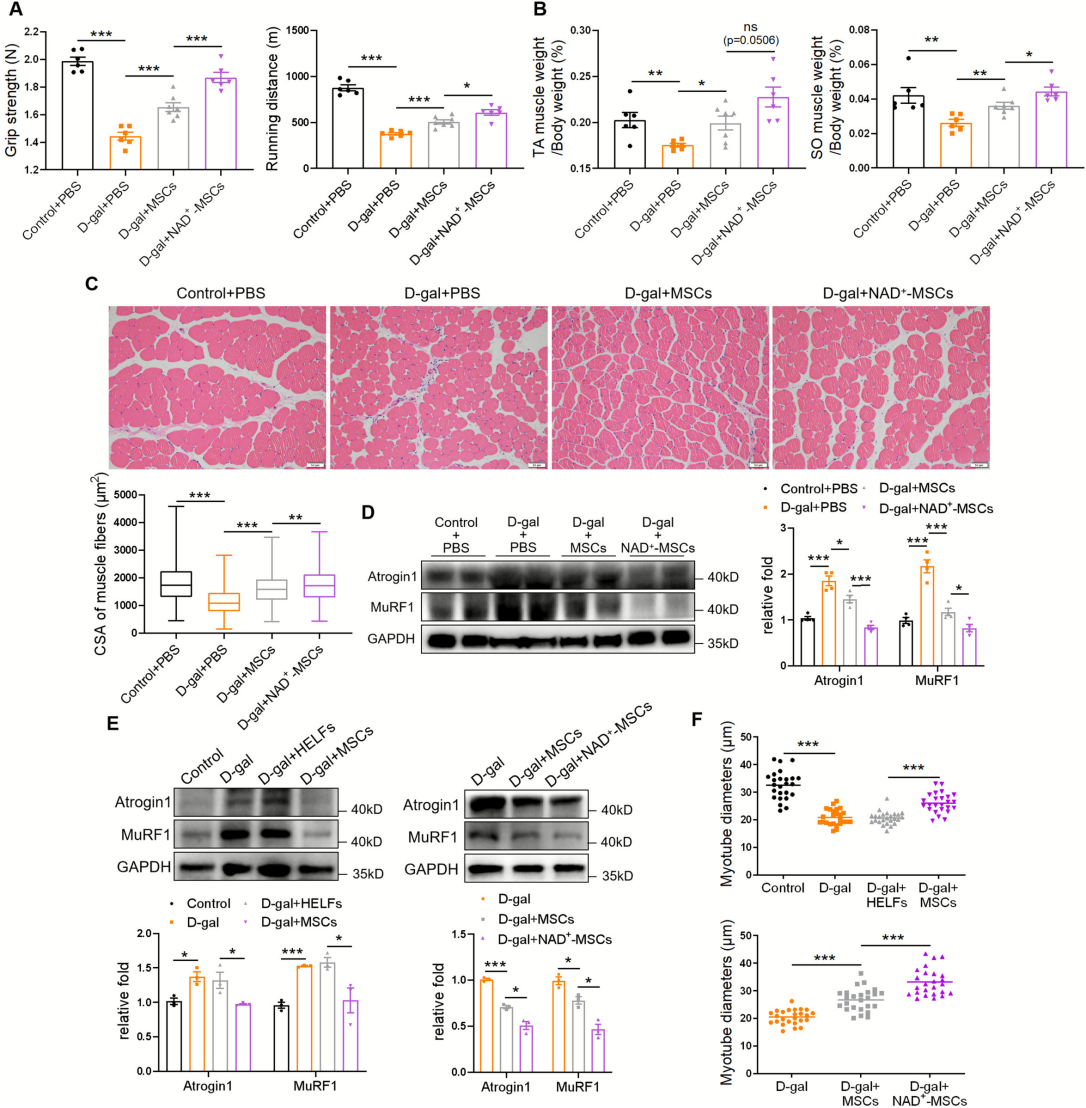
NAD^+^ pretreatment enhances the improvement effect of mesenchymal stromal cells (MSCs) on D‐gal induced muscle atrophy. (A) Grip strength and exhaustive running distance (*n* = 6–7 mice). (B) Percentage of tibialis anterior (TA) muscle weight and soleus (SO) muscle weight in body weight (*n* = 6–7 mice). (C) Haematoxylin–eosin (H&E) staining of TA muscles (scale bar, 50 μm) and cross‐sectional area (CSA) of muscle fibres (*n* = 5–6 mice, 425–575 muscle fibres for each group). (D) Western blot analysis of Atrogin 1 and MuRF1 in QUAD muscles (*n* = 4 mice). (E) Western blot analysis of Atrogin 1 and MuRF1 in C2C12 myotubes treated with D‐gal, MSCs and NAD^+^‐MSCs (*n* = 3). (F) Diameters of C2C12 myotubes treated with D‐gal, MSCs and NAD^+^‐MSCs. Quantification of bands was performed using ImageJ software. Data are presented as mean ± SEM (*p < 0.05, **p < 0.01, ***p < 0.001).

The grip strength and exhaustive running distance tests indicated a decline in muscle strength in the D‐gal + PBS group compared with that in the control + PBS group, which was elevated by MSC injection and further enhanced by NAD^+^‐MSC intervention (Figure 1A). MSCs did not influence body weight (Figure S2A) but increased TA and SO muscle mass, which was even more pronounced in the D‐gal + NAD^+^‐MSCs group (Figures 1B and S2B,C). H&E staining showed lower levels of muscle fibre CSA in the TA muscles of D‐gal + PBS mice compared with that in control + PBS mice, which were all increased by MSC treatment and further elevated by NAD^+^‐MSCs (Figure 1C). Furthermore, MSCs suppressed muscle atrophy‐associated upregulation of the E3‐ubiquitin ligases, Atrogin 1 and MuRF1, which were lower in the D‐gal + NAD^+^‐MSCs group (Figure 1D). Additionally, muscle fibrosis in D‐gal–induced mice was also decreased by MSCs and further ameliorated by NAD^+^‐MSCs (Figure S2D). Meanwhile, in C2C12 myotubes, MSC intervention downregulated D‐gal–induced Atrogin 1/MuRF1 expression and increased the myotube diameter (Figures 1E,F and S2E). NAD^+^ pretreatment further enhanced the effect of MSCs on the muscle atrophy protein Atrogin 1/MuRF1 and myotube diameter (Figures 1E,F and S2F). These results indicate that MSCs improve D‐gal–induced muscle atrophy and NAD^+^ pretreatment can further enhance the therapeutic effect of MSCs.

### 3.2 MSCs/NAD^+^‐MSCs Rescue Muscle Atrophy–Associated Impairment of the SIRT1/PGC‐1α Signalling and Mitochondrial Function

**FIGURE 2:**
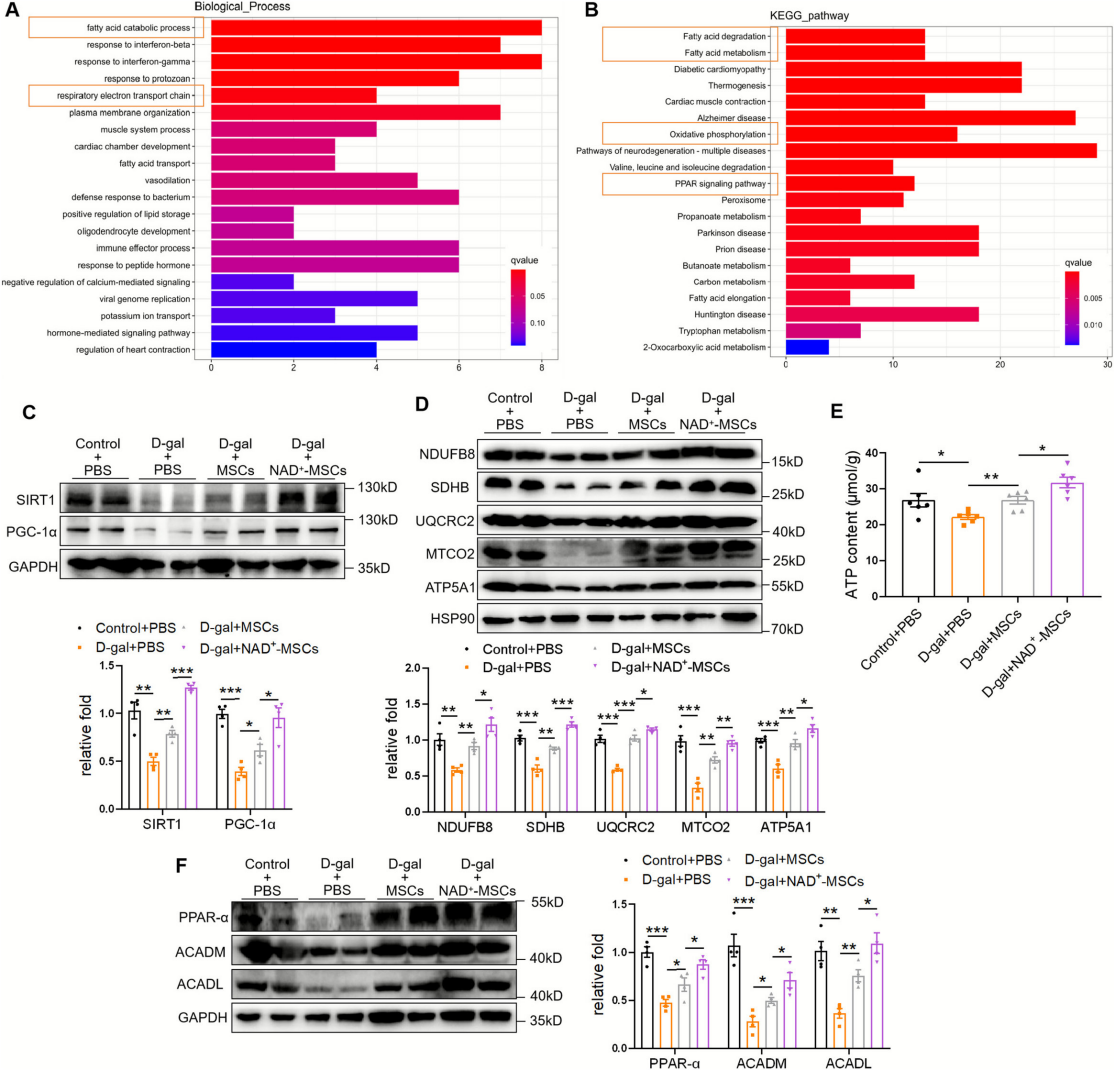
Mesenchymal stromal cells (MSCs)/NAD^+^‐MSCs rescue muscle atrophy‐associated impairment of the SIRT1/PGC‐1α signalling and mitochondrial function. (A) GO analysis of upregulated differentially expressed genes (DEGs) in biological process identified by RNA‐seq of quadriceps (QUAD) muscle from D‐gal mice with PBS or MSC treatment (*n* = 4 mice). (B) KEGG analysis of the upregulated DEGs. (C) Western blot analysis of SIRT1 and PGC‐1α in QUAD muscles (*n* = 4 mice). (D) Western blot analysis of mitochondrial complex NDUFB8, SDHB, UQCRC2, MTCO2 and ATP5A1 in QUAD muscles of D‐gal mice (*n* = 4 mice). (E) ATP content of QUAD muscles (*n* = 6 mice). (F) Western blot analysis of PPAR‐α, ACADM and ACADL in QUAD muscles (*n* = 4 mice). Quantification of bands was performed using ImageJ software. Data are presented as mean ± SEM (*p < 0.05, **p < 0.01, ***p < 0.001).

To investigate the mechanism underlying MSC‐mediated alleviation of muscle atrophy, we conducted RNA‐seq analysis of the QUAD muscle from D‐gal–exposed mice injected with PBS or MSCs. We identified 960 DEGs; 525 genes were upregulated in MSCs versus PBS, and 435 genes were downregulated (Figure S3A). GO analysis suggested that the DEGs upregulated during biological processes were enriched in fatty acid catabolic processes and the respiratory electron transport chain (Figure 2A). KEGG pathway analysis revealed that the upregulated DEGs were enriched in fatty acid degradation, fatty acid metabolism, oxidative phosphorylation and PPAR signalling (Figure 2B). Cluster of orthologous groups of proteins (COG) function classification showed that the upregulated DEGs were mainly related to energy production and lipid metabolism (Figure S3B). Meanwhile, the downregulated DEGs were mainly associated with muscle fibrosis, including complement coagulation cascades, MAPK, PI3K‐AKT and TGF‐β signalling (Figure S3C–E). Furthermore, we performed untargeted metabolomics of the QUAD muscle and found that lipid‐associated metabolites accounted for approximately one‐third of all metabolites (Figure S3F). Compared with the D‐gal + PBS group, 19 metabolites were increased and 54 metabolites were decreased in the D‐gal + MSCs group (Figure S3G), among which there were significant differences in lipid metabolites (Figure S3H). These results suggest that MSCs mainly regulate mitochondrial function and fatty acid metabolism in muscles.

SIRT1 plays a vital role in aging, mitochondrial function regulation and fatty acid oxidation [14]. Therefore, we verified the SIRT1 pathway and related indicators of mitochondrial function and fatty acid oxidation in vivo. Lower SIRT1 and downstream PGC‐1α levels were detected in the muscles of D‐gal + PBS mice than those in control + PBS mice, whereas MSC injection upregulated SIRT1/PGC‐1α levels (Figure 2C). Meanwhile, MSC intervention improved the mitochondrial structure of muscle tissue, including alleviation of swollen mitochondria and destroyed mitochondrial cristae (Figure S4A), promoted the expression of mitochondrial complexes NDUFB8, SDHB, UQCRC2, MTCO2 and ATP5A1 (Figure 2D) and increased ATP content (Figure 2E). These indicators were further improved in the NAD^+^‐MSCs group (Figure 2C–E, S4A). Moreover, MSCs decreased circulating triglyceride and low‐density lipoprotein levels and increased high**‐**density lipoprotein levels, while not influencing total cholesterol levels (Figure S4B). Additionally, MSC intervention reduced lipid deposition in muscle (Figure S4C) and promoted the expression of PPAR‐α, ACADM and ACADL, which are related to fatty acid oxidation (Figure 2F). NAD^+^‐MSCs further improved plasma lipid levels and fatty acid oxidation in muscles (Figures 2F and S4B,C). In conclusion, MSCs/NAD^+^‐MSCs upregulated muscle SIRT1 expression and improved mitochondrial function and fatty acid oxidation. These results demonstrate that MSCs activate SIRT1/PGC‐1α signalling pathways, alleviate mitochondrial dysfunction and promote fatty acid oxidation in skeletal muscles, which are enhanced by NAD^+^ pretreatment.

### 3.3 MSCs/NAD^+^‐MSCs Regulate SIRT1/PGC‐1α Signaling and Mitochondrial Function in C2C12 Myotubes

**FIGURE 3:**
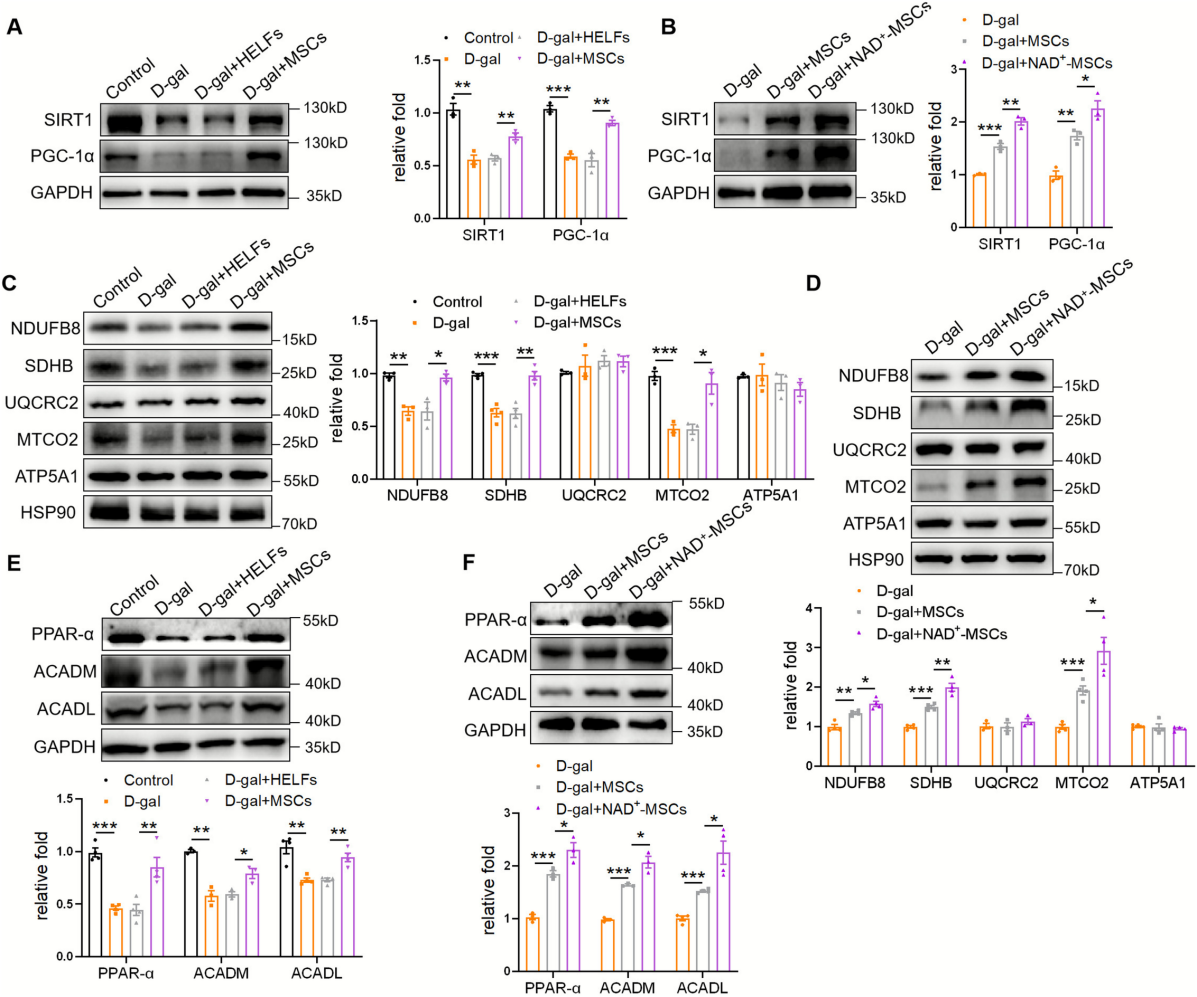
Mesenchymal stromal cells (MSCs)/NAD^+^‐MSCs regulate SIRT1/PGC‐1α signaling and mitochondrial function in C2C12 myotubes. (A) Western blot analysis of SIRT1 and PGC‐1α in C2C12 myotubes treated with D‐gal and MSCs (*n* = 3). (B) Western blot analysis of SIRT1 and PGC‐1α in C2C12 myotubes treated with MSCs and NAD^+^‐MSCs (*n* = 3). (C) Western blot analysis of mitochondrial complex NDUFB8, SDHB, UQCRC2, MTCO2 and ATP5A1 in C2C12 myotubes treated with D‐gal and MSCs (*n* = 3–4). (D) Western blot analysis of mitochondrial complex in C2C12 myotubes treated with MSCs and NAD^+^‐MSCs (*n* = 3–4). (E) Western blot analysis of PPAR‐α, ACADM and ACADL in C2C12 myotubes treated with D‐gal and MSCs (*n* = 3–4). (F) Western blot analysis of PPAR‐α, ACADM and ACADL in C2C12 myotubes treated with MSCs and NAD^+^‐MSCs (*n* = 3–4). Quantification of bands was performed using ImageJ software. Data are presented as mean ± SEM (*p < 0.05, **p < 0.01, ***p < 0.001).

To comprehensively explore the direct cell‐autonomous effects of MSCs on mitochondrial function in muscles, an in vitro C2C12‐differentiated myotube‐based model was utilised. HELFs were used as controls. MSCs increased SIRT1/PGC‐1α levels (Figure 3A) and elevated the expression of mitochondrial complexes, including NDUFB8, SDHB and MTCO2 (Figure 3C), which were further elevated by NAD^+^‐MSCs (Figure 3B,D). Moreover, MSCs/NAD^+^‐MSCs promoted the expression of PPAR‐α, ACADM and ACADL (Figure 3E,F). These results corroborate the direct cell‐autonomous effects of MSCs on myotubes and suggest that MSCs/NAD^+^‐MSCs regulate SIRT1/PGC‐1α signaling and mitochondrial function in vitro.

### 3.4 SIRT1 Mediates the Beneficial Effects of MSCs/NAD^+^‐MSCs in Myotubes

**FIGURE 4:**
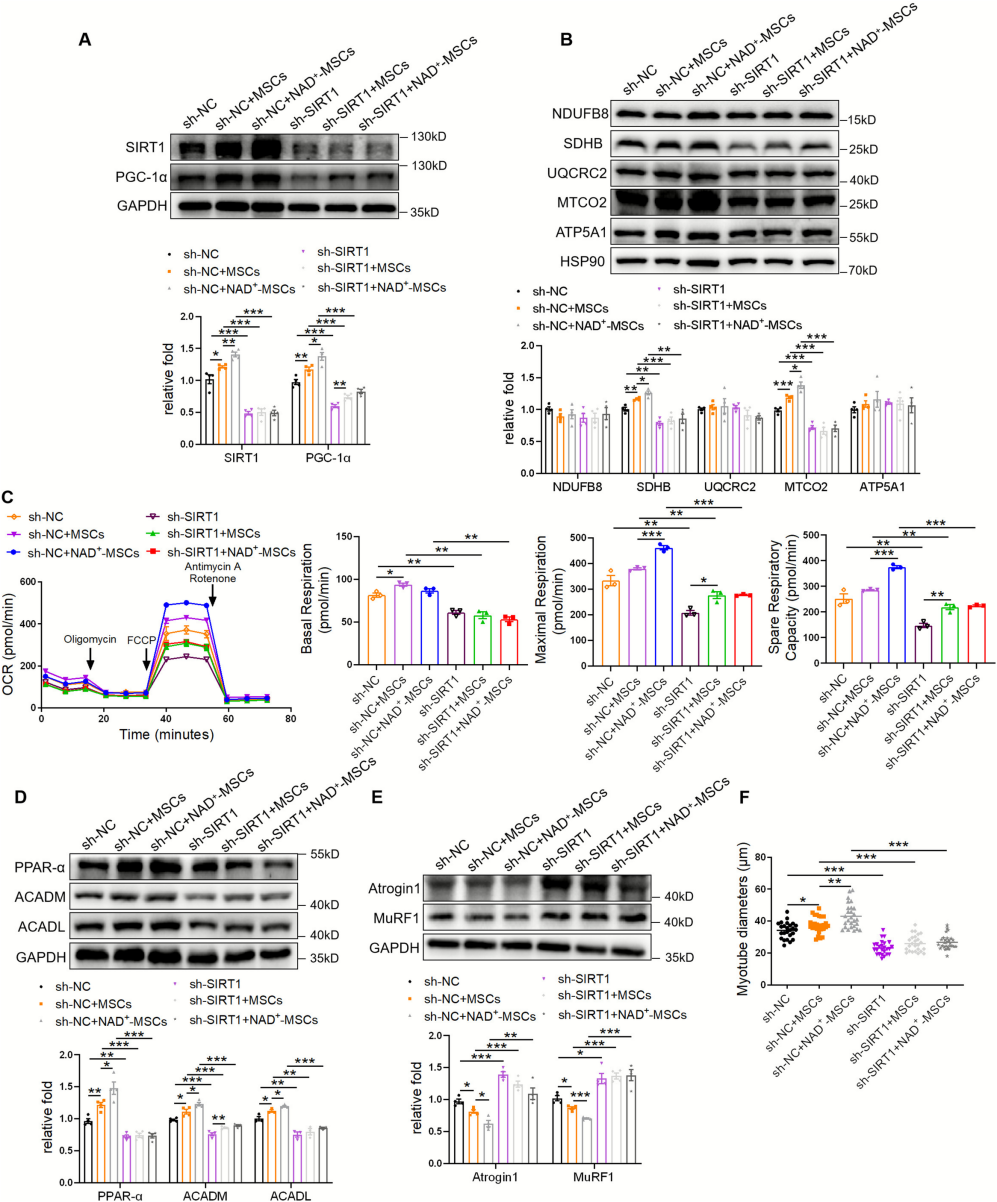
SIRT1 mediates the beneficial effects of MSCs/NAD^+^‐MSCs in myotubes. (A) Western blot analysis of SIRT1 and PGC‐1α in C2C12 myotubes transfected with *SIRT1* shRNA and treated with MSCs/NAD^+^‐MSCs (*n* = 4). (B) Western blot analysis of mitochondrial complex NDUFB8, SDHB, UQCRC2, MTCO2 and ATP5A1 (*n* = 4). (C) Seahorse analysis of OXPHOS in C2C12 myotubes transfected with *SIRT1* shRNA and treated with MSCs/NAD^+^‐MSCs, including basal respiration, maximal respiration and spare respiratory capacity (*n* = 3). (D) Western blot analysis of PPAR‐α, ACADM and ACADL (*n* = 3–4). (E) Western blot analysis of Atrogin 1 and MuRF1 (*n* = 4). (F) Diameters of C2C12 myotubes. Quantification of bands was performed using ImageJ software. Data are presented as mean ± SEM (*p < 0.05, **p < 0.01, ***p < 0.001).

To ascertain the necessity of SIRT1 in mediating the therapeutic effects of MSCs/NAD^+^‐MSCs, C2C12 myotubes were pretreated with LV‐shRNA targeting SIRT1. Western blotting showed that MSC/NAD^+^‐MSC–mediated upregulation of SIRT1/PGC‐1α signalling and mitochondrial complexes was partially weakened by sh‐SIRT1 (Figure 4A,B). Simultaneously, seahorse analysis showed that SIRT1 knockdown diminished the effects of MSCs/NAD^+^‐MSCs on oxidative phosphorylation (OXPHOS), including increased basal respiration, maximal respiration and spare respiratory capacity (Figure 4C) in myotubes. Meanwhile, MSC/NAD^+^‐MSC–mediated upregulation of PPAR‐α, ACADM and ACADL was impaired by sh‐SIRT1 (Figure 4D). Consequently, sh‐SIRT1 weakened the MSC/NAD^+^‐MSC–dependent decrease in Atrogin 1/MuRF1 levels (Figure 4E) and increase in myotube diameters (Figures 4F and S4D). These results indicate that SIRT1 mediates the beneficial effects of MSCs/NAD^+^‐MSCs in myotubes.

### 3.5 NAD^+^ Pretreatment Promotes NAMPT Secretion by MSCs

**FIGURE 5:**
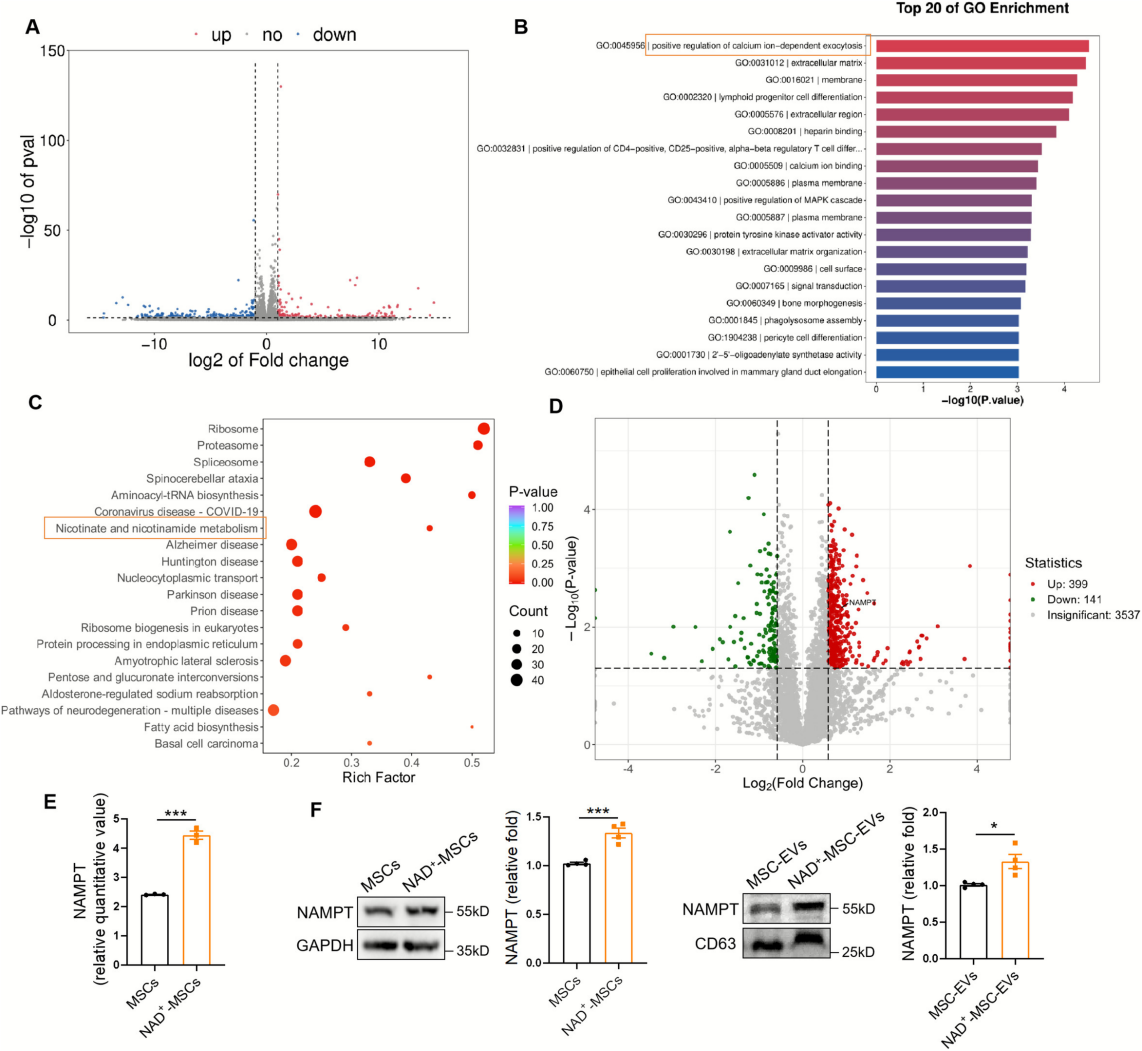
NAD^+^ pretreatment promotes NAMPT secretion by mesenchymal stromal cells (MSCs). (A) The volcano map of differentially expressed genes identified by RNA‐seq analysis of MSCs with or without NAD^+^ treatment (*n* = 3). (B) GO analysis of the enrichment pathway. (C) KEGG analysis of the enrichment pathway identified by supernatant proteomics analysis of MSCs with or without NAD^+^ treatment (*n* = 3). (D) The volcano map of differentially expressed proteins. (E) Relative quantitative value of NAMPT in supernatant of MSCs identified by proteomics (*n* = 3). (F) Western blot analysis of NAMPT in MSCs and MSC‐derived extracellular vesicles (EVs) treated with NAD^+^ (*n* = 4). Quantification of bands was performed using ImageJ software. Data are presented as mean ± SEM (*p < 0.05, ***p < 0.001).

To investigate the mechanism by which NAD^+^ enhances the therapeutic effect of MSCs, we first detected intracellular NAD^+^ levels in MSCs following NAD^+^ treatment. The results showed that NAD^+^ treatment increased intracellular NAD^+^ content and NAD^+^/NADH ratio in MSCs (Figure S5A), which suggested that NAD^+^ supplementation altered cellular NAD^+^ metabolism. Next, we performed RNA‐seq analysis of MSCs with and without NAD^+^ treatment (Figure 5A). GO enrichment analysis showed that the upregulated DEGs were mainly associated with calcium‐dependent exocytosis (Figure 5B), indicating that NAD^+^ treatment affected the paracrine effects of MSCs. Therefore, we conducted a proteomic analysis of culture supernatants derived from MSCs and NAD^+^‐MSCs. The results showed that cytoplasmic proteins exhibited the most obvious changes (Figure S5B). KEGG enrichment showed that differential proteins were enriched in nicotinate and nicotinamide metabolism (Figure 5C), and the level of NAMPT was significantly increased by NAD^+^ (Figure 5D,E). We further verified that the intracellular NAMPT expression of MSCs was upregulated after NAD^+^ treatment (Figure 5F), which laid the foundation for further mechanistic verification using siR‐NAMPT. Additionally, NAD^+^ treatment also upregulated NAMPT levels in EVs derived from MSCs (Figure 5F) without influencing the total protein content of EVs (Figure S5C), indicating that NAD^+^ increased NAMPT concentration in EVs.

### 3.6 NAD^+^ Enhances the Therapeutic Effect of MSCs on Muscle Atrophy by Promoting NAMPT Secretion

**FIGURE 6:**
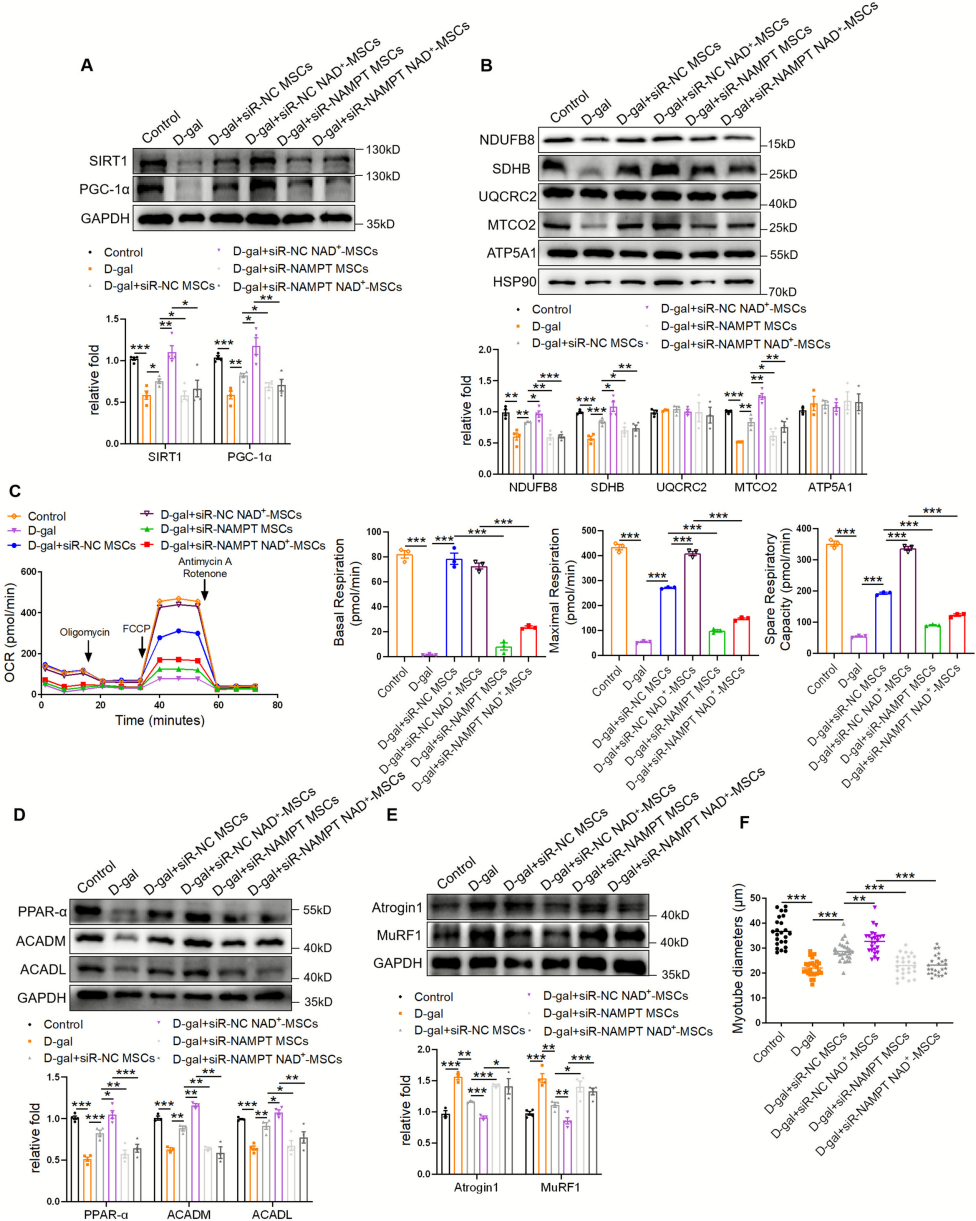
NAD^+^ enhances the therapeutic effect of mesenchymal stromal cells (MSCs) on muscle atrophy by promoting NAMPT secretion. (A) Western blot analysis of SIRT1 and PGC‐1α in C2C12 myotubes treated with D‐gal and siR‐NAMPT MSCs/NAD^+^‐MSCs (*n* = 4). (B) Western blot analysis of mitochondrial complex NDUFB8, SDHB, UQCRC2, MTCO2 and ATP5A1 (*n* = 3–4). (C) Seahorse analysis of OXPHOS in C2C12 myotubes, including basal respiration, maximal respiration and spare respiratory capacity (*n* = 3). (D) Western blot analysis of PPAR‐α, ACADM and ACADL (*n* = 3–4). (E) Western blot analysis of Atrogin 1 and MuRF1 (*n* = 3–4). (F) Diameters of C2C12 myotubes. Quantification of bands was performed using ImageJ software. Data are presented as mean ± SEM (*p < 0.05, **p < 0.01, ***p < 0.001).

To further determine whether NAD^+^ enhances the efficacy of MSCs through NAMPT, we treated MSCs/NAD^+^‐MSC with NAMPT‐targeted siRNA and co‐cultured them with D‐gal–stimulated myotubes. Western blotting showed that siR‐NAMPT effectively reduced the expression of NAMPT in MSCs, and NAD^+^‐mediated upregulation of NAMPT was eliminated by siR‐NAMPT (Figure S5D). After NAMPT knockdown, the improvement effects of MSCs/NAD^+^‐MSCs on SIRT1/PGC‐1α signalling, mitochondrial complex expression, oxidative phosphorylation and fatty acid oxidation were significantly reduced (Figure 6A–D). Consequently, siR‐NAMPT diminished the MSC/NAD^+^‐MSC–dependent decrease in Atrogin 1/MuRF1 levels (Figure 6E) and the increase in myotube diameter (Figures 6F and S5E). These results suggested that NAD^+^ enhanced the therapeutic effect of MSCs on D‐gal–induced muscle atrophy by promoting NAMPT secretion.

## 4 Discussion

**FIGURE 7:**
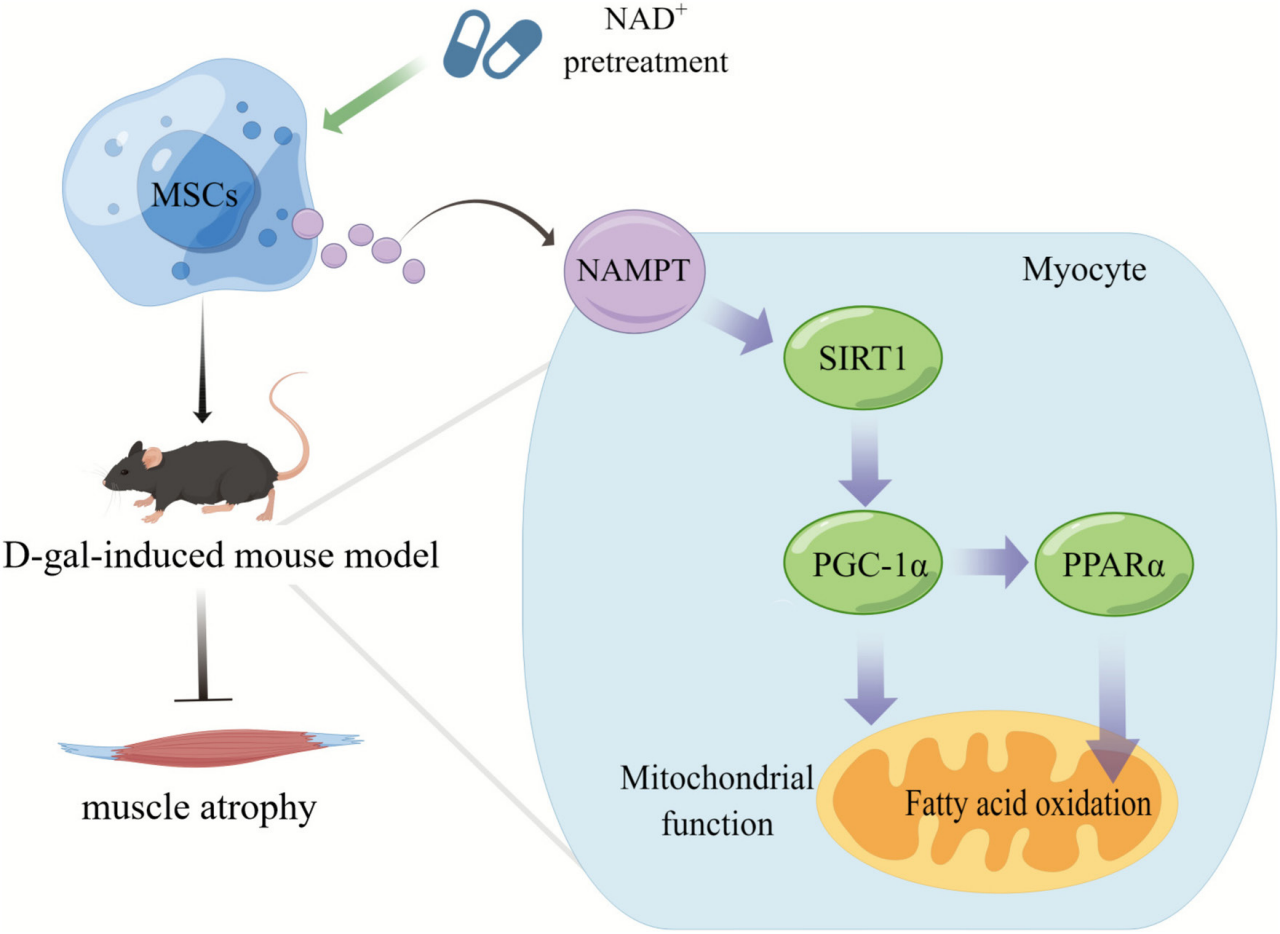
Schematic diagram of this study. NAD^+^ enhances the therapeutic effect of MSCs on D‐gal–induced muscle atrophy by promoting NAMPT secretion, which acts on the SIRT1 signalling pathway, and promotes mitochondrial function and fatty acid oxidation in skeletal muscles.

Our study elucidated that NAD^+^ enhances the therapeutic effect of MSCs on D‐gal–induced muscle atrophy by promoting NAMPT secretion, which acts on the SIRT1 signalling pathway, and promotes mitochondrial function and fatty acid oxidation in skeletal muscles (Figure 7). These results provide new insights and a theoretical basis for the clinical treatment of sarcopenia.

Sarcopenia reduces life quality and increases mortality in the elderly, but there are currently no specific strategies [4, 5]. MSCs are potential therapeutic candidates for sarcopenia [6, 15, Data S4]. Wang et al. demonstrated that clinical‐grade human umbilical cord–derived MSCs restore skeletal muscle strength and performance in two mouse models (SAMP8 mice and D‐gal–induced aging mice) by increasing the expression of extracellular matrix proteins, activating satellite cells and enhancing autophagy [6]. Takegaki et al. found that MSC injection promoted the expression of satellite cell–related genes and muscle protein synthesis and activated mTORC1 signalling [Data S4]. However, the clinical applications of MSCs are substantially limited because of their susceptibility to the surrounding environment and tendency to undergo replicative senescence during in vitro culture [19]. Therefore, it is imperative to explore ways to delay MSC aging and enhance their function. NAD^+^ is an important coenzyme involved in redox reactions [24]. NAD^+^ and its reduced form, NADH, are vital redox‐associated metabolites that mainly promote the oxidative metabolism of cells, generate energy through glycolysis and mitochondrial respiration and support cell survival and growth [20]. NAD^+^ metabolism is the basis for MSCs to exert their immune regulatory functions and plays a positive role in delaying the aging of MSCs, promoting osteogenesis and the anti‐inflammatory function of MSCs [21, 22, Data S5].

As previously mentioned, the aging model induced by D‐gal represents a comprehensive and uniform approach to accelerating the aging process systemically [Data S1]. When administered to animals, D‐gal triggers a variety of aging‐related traits, such as a reduced lifespan, heightened oxidative stress, mitochondrial DNA mutations and impaired mitochondrial function. These factors are potentially linked to the development of skeletal muscle atrophy observed during the aging process. This model is advantageous due to its simplicity, short modelling time and good repeatability [6]. Therefore, the D‐gal–induced mouse model is widely employed in studies focusing on age‐related diseases, including muscle atrophy associated with aging and anti‐aging strategies for sarcopenia [Data S2 and S3]. In the current study, we evaluated the efficacy of MSCs and NAD^+^‐pretreated MSCs (NAD^+^‐MSCs) in D‐gal–exposed mice and found that MSCs improved D‐gal–induced muscle atrophy, and NAD^+^ pretreatment further enhanced the therapeutic effect of MSCs. This provides a new method and sufficient theoretical basis for MSC empowerment to further enhance the therapeutic effect and has strong clinical significance and translational value.

To further elucidate the specific mechanisms by which NAD^+^ enhances MSC therapeutic efficacy, we first detected intracellular NAD^+^ levels and the NAD^+^/NADH ratio, which indicated that NAD^+^ supplementation altered cellular NAD^+^ metabolism. RNA‐seq of MSCs after NAD^+^ intervention showed that the upregulated DEGs were mainly associated with calcium‐dependent exocytosis, suggesting that NAD^+^ promotes the paracrine effects of MSCs. Therefore, we collected culture supernatants derived from MSCs and NAD^+^‐MSCs and conducted a proteomic analysis. The differentially expressed proteins were enriched in nicotinate and nicotinamide metabolism, among which the level of NAMPT was significantly increased by NAD^+^. NAMPT serves as the rate‐determining enzyme within the principal NAD^+^ biosynthetic cascade [25]. It exists in two distinct forms: the intracellular form, termed iNAMPT, and the extracellular form, known as eNAMPT. iNAMPT exists in the cytoplasm, nucleus and mitochondria and is mainly involved in the biosynthesis of NAD^+^. eNAMPT is involved in the NAD biosynthesis regulation and has cytokinoid functions, such as regulating cell proliferation, differentiation, migration and gene expression [26, 27]. Circulating eNAMPT levels significantly exhibited a significant age‐related decline in mice and humans [28, Data S6]. EVs containing eNAMPT promote systemic NAD^+^ biosynthesis and alleviate age‐associated functional decline in some specific target tissues, delay the age‐associated mortality rate and extend lifespan in mice [28]. To explore whether NAMPT secretion was mediated by EVs, we also extracted EVs from culture supernatants of MSCs/NAD^+^‐MSCs and found that NAD^+^ treatment upregulated NAMPT protein levels in EVs. In all, NAD^+^ supplementation promoted intracellular NAD^+^ metabolism of MSCs and increased EVs‐mediated NAMPT secretion. Subsequently, we used NAMPT‐targeted siRNA to treat MSCs/NAD^+^‐MSCs and co‐cultured them with D‐gal–stimulated myotubes. NAMPT knockdown diminished the MSC/NAD^+^‐MSC–dependent decrease in Atrogin 1/MuRF1 levels and the increase in myotube diameter, indicating that NAD^+^ enhances the therapeutic effect of MSCs on muscle atrophy by promoting NAMPT secretion.

Mitochondria represent the principal loci of lipid oxidation, an essential process in lipid catabolism and ATP synthesis, which is vital for preserving skeletal muscle homeostasis [7]. Under physiological conditions, a balance is maintained in the skeletal muscles between type I muscle fibres, which use fatty acids as metabolic substrates, and type II muscle fibres, which rely on glucose as a metabolic substrate [29]. However, in the aging state, type I fibres, which depend on mitochondrial oxidative phosphorylation, remain unchanged, while the number and size of type II skeletal muscle fibres, which rely on glycolysis, gradually decrease. This leads to a shift in skeletal muscle metabolism, primarily from glycolytic to oxidative pathways, with an increased demand for functional mitochondria [8, 9]. Mitochondrial dysfunction is a major hallmark of aging [30] and significantly contributes to the age‐related decline of skeletal muscle [31, 32, Data S7]. Diminished activity across all respiratory complexes within the mitochondria directly leads to reduced muscle mass, decreased muscle strength and impaired walking speed [31, 33]. This remodelling of muscle fibres and the mitochondrial dysfunction caused by aging further interfere with fatty acid β‐oxidation, leading to lipid accumulation in muscles and impairing muscle regeneration [10]. Transcriptomics and metabolomics have shown that skeletal muscles of patients with sarcopenia exhibit significant metabolic changes, including fatty acid metabolism and the tricarboxylic acid cycle [11]. To investigate the mechanism underlying MSC‐mediated alleviation of muscle atrophy, we performed RNA‐seq and untargeted metabolomics of QUAD muscles from D‐gal–exposed mice with PBS or MSC injections. These results suggest that the DEGs were mainly enriched in oxidative phosphorylation and fatty acid metabolism. After validation in vivo and in vitro, we confirmed that MSCs elevated the expression of mitochondrial respiratory complexes and fatty acid oxidation indicators, which were further improved by NAD^+^‐MSCs. Our results indicate that mitochondrial function and fatty acid oxidation play crucial roles in the improvement of D‐gal–induced muscle atrophy by MSCs/NAD^+^‐MSCs.

SIRT1 is a promising target for the treatment and prognosis of sarcopenia [13]. Skeletal muscle exhibits pronounced energy metabolic activity, and SIRT1 is sensitive to the cellular redox environment. Therefore, SIRT1 may influence skeletal muscle mitochondrial function and energy metabolism, and its activation shows significant promise for mitigating sarcopenia [14]. PGC‐1α exhibits a strong association with sarcopenia and plays a pivotal role in mitochondrial biogenesis [34], which was reduced in aged skeletal muscle [35]. Within skeletal muscle, the SIRT1/PGC‐1α signalling pathway is thought to collaboratively mitigate mitochondrial dysfunction and alleviate sarcopenia [36]. By enhancing mitochondrial biogenesis through PGC‐1α and PPAR‐α–mediated fatty acid oxidation, SIRT1 established a critical link between energy metabolism and age‐related muscle dysfunction [14, 37], in which its activity was regulated by NAMPT [38]. Our previous study reported that MSC‐derived exosomes alleviated diabetes‐related muscle atrophy via SIRT1‐mediated mitochondrial function [18]. In this study, we confirmed that MSCs promoted SIRT1 signalling in skeletal muscles and C2C12 myotubes, which were further elevated in the NAD^+^‐MSC group. SIRT1 knockdown weakened the effects of MSCs/NAD^+^‐MSCs on mitochondrial function, fatty acid oxidation and myotube atrophy. Consequently, SIRT1‐mediated mitochondrial homeostasis and lipid metabolism play key roles in the amelioration of muscle atrophy induced by MSCs/NAD^+^‐MSCs.

However, there were some limitations in our study. First, while the D‐gal–induced mouse model exhibited certain aging‐like features, it did not fully replicate the multifactorial, systemic nature of age‐related decline in skeletal muscle mass and function. In future research, we will continue to explore the effect of NAD^+^‐pretreated MSCs on sarcopenia using a natural aging mouse model. Next, we only used male mice in this study and not female mice. One study has shown that aging has a more severe impact on the skeletal muscle mitochondrial proteome in females compared to males, as males maintained their testosterone levels throughout aging [39]. Therefore, it is highly necessary to continue exploring the therapeutic effect of MSCs/NAD^+^‐MSCs on muscle atrophy in female mice. Furthermore, we still need to assess the therapeutic effect of MSCs/NAD^+^‐MSCs in the clinical application of age‐related muscle atrophy to provide scientific foundations for clinical treatments. In addition, despite focusing on NAD^+^, future studies will investigate other pretreatment strategies to enhance the efficacy of MSCs.

## Funding

This work was supported by Noncommunicable Chronic Diseases–National Science and Technology Major Project (2023ZD0507700, 2023ZD0507702), the National Natural Science Foundation of China (82501893, 82203191), the Natural Science Foundation of Shandong Province (ZR2024QH510), the Major Basic Research Project of the Shandong Provincial Natural Science Foundation (ZR2022ZD15) and the cross‐breed fund of the Second Qilu Hospital of Shandong University (2023JX27).

## Ethics Statement

The authors certify that they complied with the ethical guidelines for authorship and publication in the Journal of Cachexia, Sarcopenia and Muscle [40]. All animal studies were approved by the Animal Ethics Committee of the Qilu Hospital of Shandong University and were performed in accordance with the ethical standards of the 1964 Declaration of Helsinki and its later amendments. The manuscript does not contain any clinical studies or patient data.

## Conflicts of Interest

The authors declare no conflicts of interest.

## Supporting information


**Figure S1:** **Flowchart of the animal experiment and identification of MSCs/NAD** ^**+**^ **‐MSCs.** (A) Flowchart of the animal experiment. (B) Flow cytometry analysis of MSCs/NAD^+^‐MSCs markers CD105, CD73, CD34 and HLA‐DR. (C) Oil red O staining for adipogenic differentiation ability of MSCs/NAD^+^‐MSCs (scale bar, 20 μm). (D) Alizarin red S staining for osteogenic differentiation ability of MSCs/NAD^+^‐MSCs (scale bar, 50 μm). (E) RT‐qPCR analysis of p16, p21, IL‐1β and IL‐6 mRNA levels in MSCs/NAD^+^‐MSCs (*n* = 3). Data are presented as mean ± SEM (**p < 0.01, ***p < 0.001).
**Figure S2: NAD** ^**+**^ **pretreatment enhances the improvement effect of mesenchymal stromal cells (MSCs) on D‐gal induced muscle atrophy**. (A) Body weight (*n* = 6–7 mice). (B) Tibialis anterior (TA) muscle weight (*n* = 6–7 mice). (C) Soleus (SO) muscle weight (*n* = 6–7 mice). (D) Sirius red and Masson staining of TA muscles (scale bar, 50 μm). (E) Representative images of C2C12 myotubes treated with D‐gal and MSCs (scale bar, 50 μm). (F) Representative images of C2C12 myotubes treated with MSCs and NAD^+^‐MSCs (scale bar, 50 μm). Data are presented as mean ± SEM. (*p < 0.05, **p < 0.01).
**Figure S3: RNA‐seq and metabolomics analysis of quadriceps (QUAD) muscle from D‐gal mice with PBS or MSC treatment.** (A) The volcano map of differentially expressed genes identified by RNA‐seq analysis of quadriceps (QUAD) muscle from D‐gal mice with PBS or MSC treatment (*n* = 4 mice). (B) COG function classification of consensus sequence. (C) GO analysis of the down‐regulated DEGs in cellular component. (D, E) KEGG analysis of the down‐regulated DEGs. (F) Super class of metabolites identified by untargeted metabolomics of quadriceps (QUAD) muscle from D‐gal mice with PBS or MSC treatment (*n* = 6 mice). (G) The volcano map of differentially expressed metabolites. (H) The chord plot of differentially expressed metabolites.
**Figure S4: Mesenchymal stromal cells (MSCs)/NAD** ^**+**^ **‐MSCs rescue muscle atrophy‐associated impairment of mitochondrial function and lipid deposition through SIRT1**. (A) Transmission electron microscopy (TEM) images of intermyofibrillar (IMF) mitochondria in tibialis anterior (TA) muscles (scale bar, 1.2 μm). (B) Plasma triglyceride (TG), total cholesterol (TC), low‐density lipoprotein (LDL) and high**‐**density lipoprotein (HDL) levels of mice (*n* = 5–6 mice). (C) Oil red O staining of QUAD muscles (scale bar, 50 μm) and quantification of lipid content (*n* = 3 mice). (D) Representative images of C2C12 myotubes transfected with *SIRT1* shRNA and treated with MSCs/NAD^+^‐MSCs (scale bar, 50 μm). Data are presented as mean ± SEM. (*p < 0.05, **p < 0.01).
**Figure S5: NAD** ^**+**^ **enhances the therapeutic effect of mesenchymal stromal cells (MSCs) on muscle atrophy by promoting NAMPT secretion.** (A) Intracellular NAD^+^ content and NAD^+^/NADH ratio of MSCs with or without NAD^+^ treatment (*n* = 3). (B) Subcellular localization of protein component identified by supernatant proteomics analysis of MSCs with or without NAD^+^ treatment (*n* = 3). (C) The total protein content of EVs derived from MSCs with or without NAD^+^ treatment (*n* = 4). (D) Western blot analysis of NAMPT in MSCs transfected with NAMPT siRNA and treated with NAD^+^ (*n* = 3). Quantification of bands was performed using ImageJ software. (E) Representative images of C2C12 myotubes treated with D‐gal and siR‐NAMPT MSCs/NAD^+^‐MSCs (scale bar, 50 μm). Data are presented as mean ± SEM. (*p < 0.05, **p < 0.01, ***p < 0.001).

**Data S1:** Supplementary References.

**Data S2:** Supplementary Information.

## Data Availability

The RNA‐seq data can be available in the Gene Expression Omnibus (GEO) datasets (GSE304351). Other data generated or analysed during this study are included in this published article.

## References

[1] Y. Wang , Y. Zhang , N. E. Lane , et al., “Population‐Based Metagenomics Analysis Reveals Altered Gut Microbiome in Sarcopenia: Data From the Xiangya Sarcopenia Study,” Journal of Cachexia, Sarcopenia and Muscle 13, no. 5 (2022): 2340–2351, 10.1002/jcsm.13037. 35851765 PMC9530518

[2] J. Zanker , M. Sim , K. Anderson , et al., “Consensus Guidelines for Sarcopenia Prevention, Diagnosis and Management in Australia and New Zealand,” Journal of Cachexia, Sarcopenia and Muscle 14, no. 1 (2023): 142–156, 10.1002/jcsm.13115. 36349684 PMC9891980

[3] S. Zhou , L. Li , S. Li , H. Si , L. Wu , and B. Shen , “The Negative Impacts of Sarcopenia on Primary Total Knee Arthroplasty Under the Enhanced Recovery After Surgery Protocol,” Orthopaedic Surgery 16, no. 5 (2024): 1160–1167, 10.1111/os.14053. 38556481 PMC11062878

[4] E. Benz , A. Pinel , C. Guillet , et al., “Sarcopenia and Sarcopenic Obesity and Mortality Among Older People,” JAMA Network Open 7, no. 3 (2024): e243604, 10.1001/jamanetworkopen.2024.3604. 38526491 PMC10964118

[5] A. A. Sayer , R. Cooper , H. Arai , et al., “Sarcopenia,” Nature Reviews. Disease Primers 10, no. 1 (2024): 68, 10.1038/s41572-024-00550-w. 39300120

[6] C. Wang , B. Zhao , J. Zhai , et al., “Clinical‐Grade Human Umbilical Cord–Derived Mesenchymal Stem Cells Improved Skeletal Muscle Dysfunction in Age‐Associated Sarcopenia Mice,” Cell Death & Disease 14, no. 5 (2023): 321, 10.1038/s41419-023-05843-8. 37173309 PMC10182022

[7] D. A. Hood , J. M. Memme , A. N. Oliveira , and M. Triolo , “Maintenance of Skeletal Muscle Mitochondria in Health, Exercise, and Aging,” Annual Review of Physiology 81 (2019): 19–41, 10.1146/annurev-physiol-020518-114310. 30216742

[8] D. Sebastián , E. Sorianello , J. Segalés , et al., “Mfn2 Deficiency Links Age‐Related Sarcopenia and Impaired Autophagy to Activation of an Adaptive Mitophagy Pathway,” EMBO Journal 35, no. 15 (2016): 1677–1693, 10.15252/embj.201593084. 27334614 PMC4969577

[9] A. R. Palla , M. Ravichandran , Y. X. Wang , et al., “Inhibition of Prostaglandin‐Degrading Enzyme 15‐PGDH Rejuvenates Aged Muscle Mass and Strength,” Science 371 (2021):eabc8059, 10.1126/science.abc8059. 33303683 PMC7938328

[10] J. P. Gumucio , A. H. Qasawa , P. J. Ferrara , et al., “Reduced Mitochondrial Lipid Oxidation Leads to Fat Accumulation in Myosteatosis,” FASEB Journal 33, no. 7 (2019): 7863–7881, 10.1096/fj.201802457RR. 30939247 PMC6593892

[11] X. Zuo , R. Zhao , M. Wu , et al., “Multi‐Omic Profiling of Sarcopenia Identifies Disrupted Branched‐Chain Amino Acid Catabolism as a Causal Mechanism and Therapeutic Target,” Nature Aging 5, no. 3 (2025): 419–436, 10.1038/s43587-024-00797-8. 39910243

[12] M. J. Han and S. Y. Choung , “ *Codonopsis lanceolata* Ameliorates Sarcopenic Obesity via Recovering PI3K/Akt Pathway and Lipid Metabolism in Skeletal Muscle,” Phytomedicine 96 (2022): 153877, 10.1016/j.phymed.2021.153877. 35026519

[13] Z. Zhang , Q. Guo , Z. Yang , et al., “ *Bifidobacterium adolescentis* –Derived Nicotinic Acid Improves Host Skeletal Muscle Mitochondrial Function to Ameliorate Sarcopenia,” Cell Reports 44, no. 2 (2025): 115265, 10.1016/j.celrep.2025.115265. 39908139

[14] L. Yang , D. Liu , S. Jiang , et al., “SIRT1 Signaling Pathways in Sarcopenia: Novel Mechanisms and Potential Therapeutic Targets,” Biomedicine & Pharmacotherapy 177 (2024): 116917, 10.1016/j.biopha.2024.116917. 38908209

[15] Y. Kono , “Preparation of Magnetized Mesenchymal Stem Cells Using Magnetic Liposomes to Enhance Their Retention in Targeted Tissue—Evaluation of Retention and Anti‐Inflammatory Efficiency in Skeletal Muscle,” Yakugaku Zasshi: Journal of the Pharmaceutical Society of Japan 142, no. 11 (2022): 1145–1151, 10.1248/yakushi.22-00132. 36328443

[16] X. Liu , X. Li , W. Zhu , et al., “Exosomes From Mesenchymal Stem Cells Overexpressing MIF Enhance Myocardial Repair,” Journal of Cellular Physiology 235, no. 11 (2020): 8010–8022, 10.1002/jcp.29456. 31960418

[17] Y. Yuan , M. Shi , L. Li , et al., “Mesenchymal Stem Cell–Conditioned Media Ameliorate Diabetic Endothelial Dysfunction by Improving Mitochondrial Bioenergetics via the Sirt1/AMPK/PGC‐1α Pathway,” Clinical Science (London, England) 130, no. 23 (2016): 2181–2198, 10.1042/CS20160235. 27613156

[18] J. Song , M. M. Yang , L. Q. Xia , et al., “Aptamer‐Conjugated Exosomes Ameliorate Diabetes‐Induced Muscle Atrophy by Enhancing SIRT1/FoxO1/3a‐Mediated Mitochondrial Function,” Journal of Cachexia, Sarcopenia and Muscle 16, no. 1 (2025): e13717, 10.1002/jcsm.13717. 39871746 PMC11773161

[19] P. F. Wong , M. Dharmani , and T. S. Ramasamy , “Senotherapeutics for Mesenchymal Stem Cell Senescence and Rejuvenation,” Drug Discovery Today 28, no. 1 (2023): 103424, 10.1016/j.drudis.2022.103424. 36332835

[20] A. J. Covarrubias , R. Perrone , A. Grozio , and E. Verdin , “NAD(+) Metabolism and Its Roles in Cellular Processes During Ageing,” Nature Reviews. Molecular Cell Biology 22, no. 2 (2021): 119–141, 10.1038/s41580-020-00313-x. 33353981 PMC7963035

[21] J. Fang , P. Hou , S. Liu , et al., “NAD+ Salvage Governs the Immunosuppressive Capacity of Mesenchymal Stem Cells,” Cellular & Molecular Immunology 20, no. 10 (2023): 1171–1185, 10.1038/s41423-023-01073-2. 37580400 PMC10541442

[22] J. Song , J. Li , F. Yang , et al., “Nicotinamide Mononucleotide Promotes Osteogenesis and Reduces Adipogenesis by Regulating Mesenchymal Stromal Cells via the SIRT1 Pathway in Aged Bone Marrow,” Cell Death & Disease 10, no. 5 (2019): 336, 10.1038/s41419-019-1569-2. 31000692 PMC6472410

[23] J. Song , J. Liu , C. Cui , et al., “Mesenchymal Stromal Cells Ameliorate Diabetes‐Induced Muscle Atrophy Through Exosomes by Enhancing AMPK/ULK1‐Mediated Autophagy,” Journal of Cachexia, Sarcopenia and Muscle 14, no. 2 (2023): 915–929, 10.1002/jcsm.13177. 36708027 PMC10067482

[24] M. E. Migaud , M. Ziegler , and J. A. Baur , “Regulation of and Challenges in Targeting NAD(+) Metabolism,” Nature Reviews. Molecular Cell Biology 25, no. 10 (2024): 822–840, 10.1038/s41580-024-00752-w. 39026037 PMC12456757

[25] J. Cheng , J. Zhang , S. He , M. Li , G. Dong , and C. Sheng , “Photoswitchable PROTACs for Reversible and Spatiotemporal Regulation of NAMPT and NAD,” Angewandte Chemie (International Ed. in English) 63, no. 12 (2024): e202315997, 10.1002/anie.202315997. 38282119

[26] A. Garten , S. Schuster , M. Penke , T. Gorski , T. de Giorgis , and W. Kiess , “Physiological and Pathophysiological Roles of NAMPT and NAD Metabolism,” Nature Reviews. Endocrinology 11 (2015): 535–546, 10.1038/nrendo.2015.117. 26215259

[27] J. R. Revollo , A. Körner , K. F. Mills , et al., “Nampt/PBEF/Visfatin Regulates Insulin Secretion in β Cells as a Systemic NAD Biosynthetic Enzyme,” Cell Metabolism 6 (2007): 363–375, 10.1016/j.cmet.2007.09.003. 17983582 PMC2098698

[28] M. Yoshida , A. Satoh , J. B. Lin , et al., “Extracellular Vesicle–Contained eNAMPT Delays Aging and Extends Lifespan in Mice,” Cell Metabolism 30, no. 2 (2019): 329–342.e5, 10.1016/j.cmet.2019.05.015. 31204283 PMC6687560

[29] O. Horwath , L. Cornet , H. Strömlind , et al., “Endurance Exercise With Reduced Muscle Glycogen Content Influences Substrate Utilization and Attenuates Acute mTORC1‐ and Autophagic Signaling in Human Type I and Type II Muscle Fibers,” Skeletal Muscle 15, no. 1 (2025): 9, 10.1186/s13395-025-00377-3. 40128889 PMC11934587

[30] C. López‐Otín , M. A. Blasco , L. Partridge , M. Serrano , and G. Kroemer , “Hallmarks of Aging: An Expanding Universe,” Cell 186 (2023): 243–278, 10.1016/j.cell.2013.05.039. 36599349

[31] E. Migliavacca , S. K. H. Tay , H. P. Patel , et al., “Mitochondrial Oxidative Capacity and NAD^+^ Biosynthesis Are Reduced in Human Sarcopenia Across Ethnicities,” Nature Communications 10 (2019): 5808, 10.1038/s41467-019-13694-1. PMC6925228 31862890

[32] G. Gherardi , A. Weiser , F. Bermont , et al., “Mitochondrial Calcium Uptake Declines During Aging and Is Directly Activated by Oleuropein to Boost Energy Metabolism and Skeletal Muscle Performance,” Cell Metabolism 37, no. 2 (2025): 477–495.e11, 10.1016/j.cmet.2024.10.021. 39603237

[33] A. C. Zane , D. A. Reiter , M. Shardell , et al., “Muscle Strength Mediates the Relationship Between Mitochondrial Energetics and Walking Performance,” Aging Cell 16 (2017): 461–468, 10.1111/acel.12568. 28181388 PMC5418194

[34] S. Kong , B. Cai , and Q. Nie , “PGC‐1alpha Affects Skeletal Muscle and Adipose Tissue Development by Regulating Mitochondrial Biogenesis,” Molecular Genetics and Genomics 297, no. 3 (2022): 621–633, 10.1007/s00438-022-01878-2. 35290519

[35] A. M. Joseph , P. J. Adhihetty , T. W. Buford , et al., “The Impact of Aging on Mitochondrial Function and Biogenesis Pathways in Skeletal Muscle of Sedentary High‐ and Low‐Functioning Elderly Individuals,” Aging Cell 11, no. 5 (2012): 801–809, 10.1111/j.1474-9726.2012.00844.x. 22681576 PMC3444680

[36] L. Tian , W. Cao , R. Yue , et al., “Pretreatment With Tilianin Improves Mitochondrial Energy Metabolism and Oxidative Stress in Rats With Myocardial Ischemia/Reperfusion Injury via AMPK/SIRT1/PGC‐1 Alpha Signaling Pathway,” 139, no. 4 (2019): 352–360, 10.1016/j.jphs.2019.02.008. 30910451

[37] E. Q. Toyama , S. Herzig , J. Courchet , et al., “AMP‐Activated Protein Kinase Mediates Mitochondrial Fission in Response to Energy Stress,” Science 351, no. 6270 (2016): 275–281, 10.1126/science.aab4138. 26816379 PMC4852862

[38] Y. Jiang , Y. Wang , G. Chen , et al., “Nicotinamide Metabolism Face‐Off Between Macrophages and Fibroblasts Manipulates the Microenvironment in Gastric Cancer,” Cell Metabolism 36, no. 8 (2024): 1806–1822.e11, 10.1016/j.cmet.2024.05.013. 38897198

[39] A. Moreira‐Pais , R. Vitorino , C. Sousa‐Mendes , et al., “Mitochondrial Remodeling Underlying Age‐Induced Skeletal Muscle Wasting: Let's Talk About Sex,” Free Radical Biology & Medicine 218 (2024): 68–81, 10.1016/j.freeradbiomed.2024.04.005. 38574975

[40] S. von Haehling , A. J. S. Coats , and S. D. Anker , “Ethical Guidelines for Publishing in the *Journal of Cachexia, Sarcopenia and Muscle*: Update 2021,” Journal of Cachexia, Sarcopenia and Muscle 12, no. 6 (2021): 2259–2261, 10.1002/jcsm.12899. 34904399 PMC8718061

