# Supplementary material for: NAD+ Enhanced Mesenchymal Stromal Cells Effect on Muscle Atrophy by Improving SIRT1‐Mediated Mitochondrial Function via NAMPT
Source: J Cachexia Sarcopenia Muscle. 2025 Dec 12;16(6):e70142. doi: 10.1002/jcsm.70142 (PMC12699140; doi:10.1002/jcsm.70142)
Supplement: Supplementary file 1 — Figure S1: Flowchart of the animal experiment and identification of MSCs/NAD + ‐MSCs. (A) Flowchart of the animal experiment. (B) Flow cytometry analysis of MSCs/NAD+‐MSCs markers CD105, CD73, CD34 and HLA‐DR. (C) Oil red O staining for adipogenic differentiation ability of MSCs/NAD+‐MSCs (scale bar, 20 μm). (D) Alizarin red S staining for osteogenic differentiation ability of MSCs/NAD+‐MSCs (scale bar, 50 μm). (E) RT‐qPCR analysis of p16, p21, IL‐1β and IL‐6 mRNA levels in MSCs/NAD+‐MSCs (n = 3). Data are presented as mean ± SEM (**p < 0.01, ***p < 0.001). Figure S2: NAD + pretreatment enhances the improvement effect of mesenchymal stromal cells (MSCs) on D‐gal induced muscle atrophy. (A) Body weight (n = 6–7 mice). (B) Tibialis anterior (TA) muscle weight (n = 6–7 mice). (C) Soleus (SO) muscle weight (n = 6–7 mice). (D) Sirius red and Masson staining of TA muscles (scale bar, 50 μm). (E) Representative images of C2C12 myotubes treated with D‐gal and MSCs (scale bar, 50 μm). (F) Representative images of C2C12 myotubes treated with MSCs and NAD+‐MSCs (scale bar, 50 μm). Data are presented as mean ± SEM. (*p < 0.05, **p < 0.01). Figure S3: RNA‐seq and metabolomics analysis of quadriceps (QUAD) muscle from D‐gal mice with PBS or MSC treatment. (A) The volcano map of differentially expressed genes identified by RNA‐seq analysis of quadriceps (QUAD) muscle from D‐gal mice with PBS or MSC treatment (n = 4 mice). (B) COG function classification of consensus sequence. (C) GO analysis of the down‐regulated DEGs in cellular component. (D, E) KEGG analysis of the down‐regulated DEGs. (F) Super class of metabolites identified by untargeted metabolomics of quadriceps (QUAD) muscle from D‐gal mice with PBS or MSC treatment (n = 6 mice). (G) The volcano map of differentially expressed metabolites. (H) The chord plot of differentially expressed metabolites. Figure S4: Mesenchymal stromal cells (MSCs)/NAD + ‐MSCs rescue muscle atrophy‐associated impairment of mitochondrial function [file JCSM-16-e70142-s003.docx]

**Supplementary figures**

**
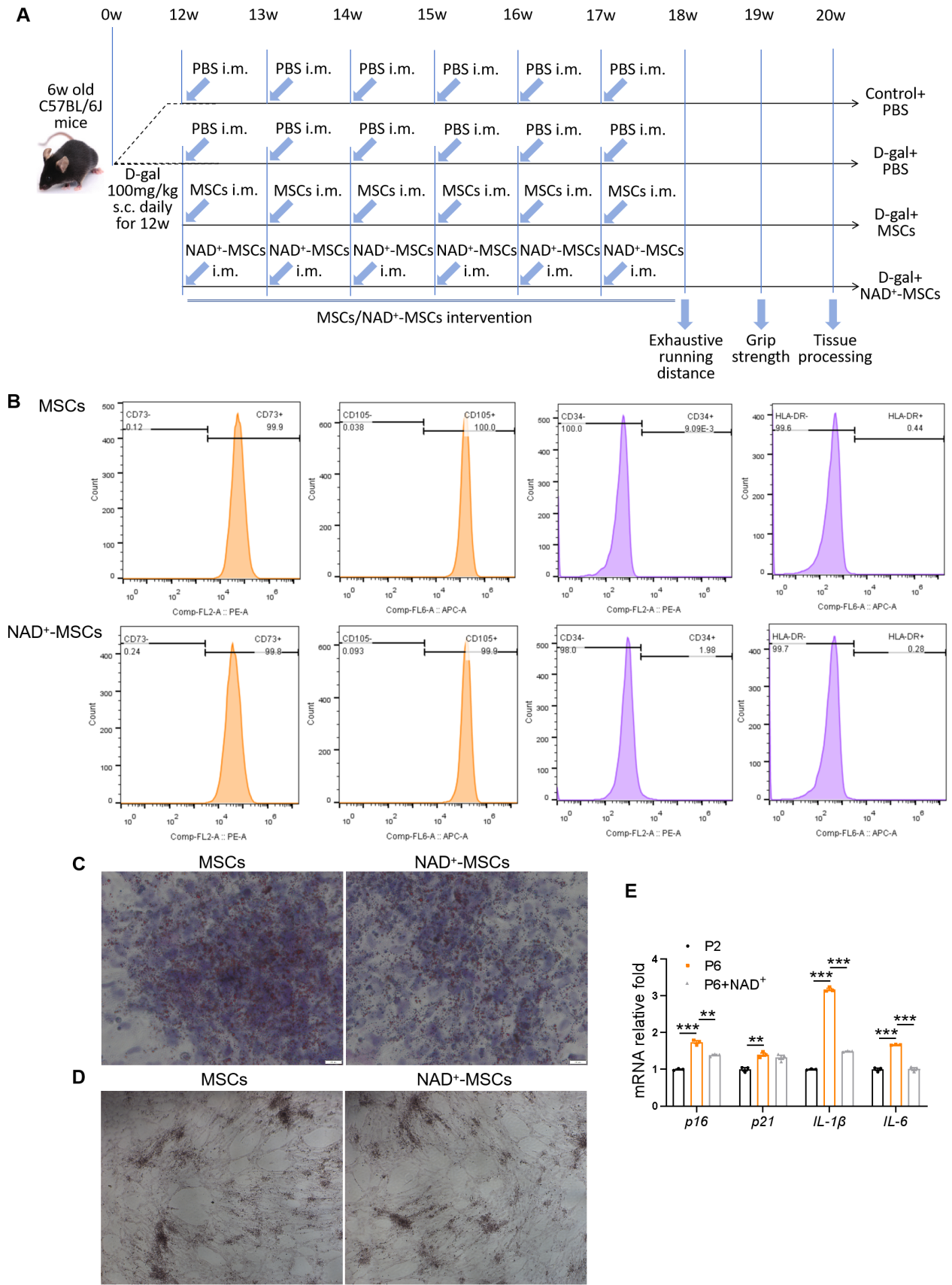
Supplementary Figure 1.** **Flow chart of the animal experiment and identification of MSCs/NAD^+^-MSCs.** (A) Flow chart of the animal experiment. (B) Flow cytometry analysis of MSCs/NAD^+^-MSCs markers CD105, CD73, CD34, and HLA‐DR. (C) Oil Red O staining for adipogenic differentiation ability of MSCs/NAD^+^-MSCs (scale bar, 20 μm). (D) Alizarin Red S staining for osteogenic differentiation ability of MSCs/NAD^+^-MSCs (Scale bar, 50 μm). (E) RT‐qPCR analysis of *p16, p21, IL-1β* and *IL-6* mRNA levels in MSCs/NAD^+^-MSCs (n=3). Data are presented as mean ± SEM. (***P* < 0.01, ****P* < 0.001)


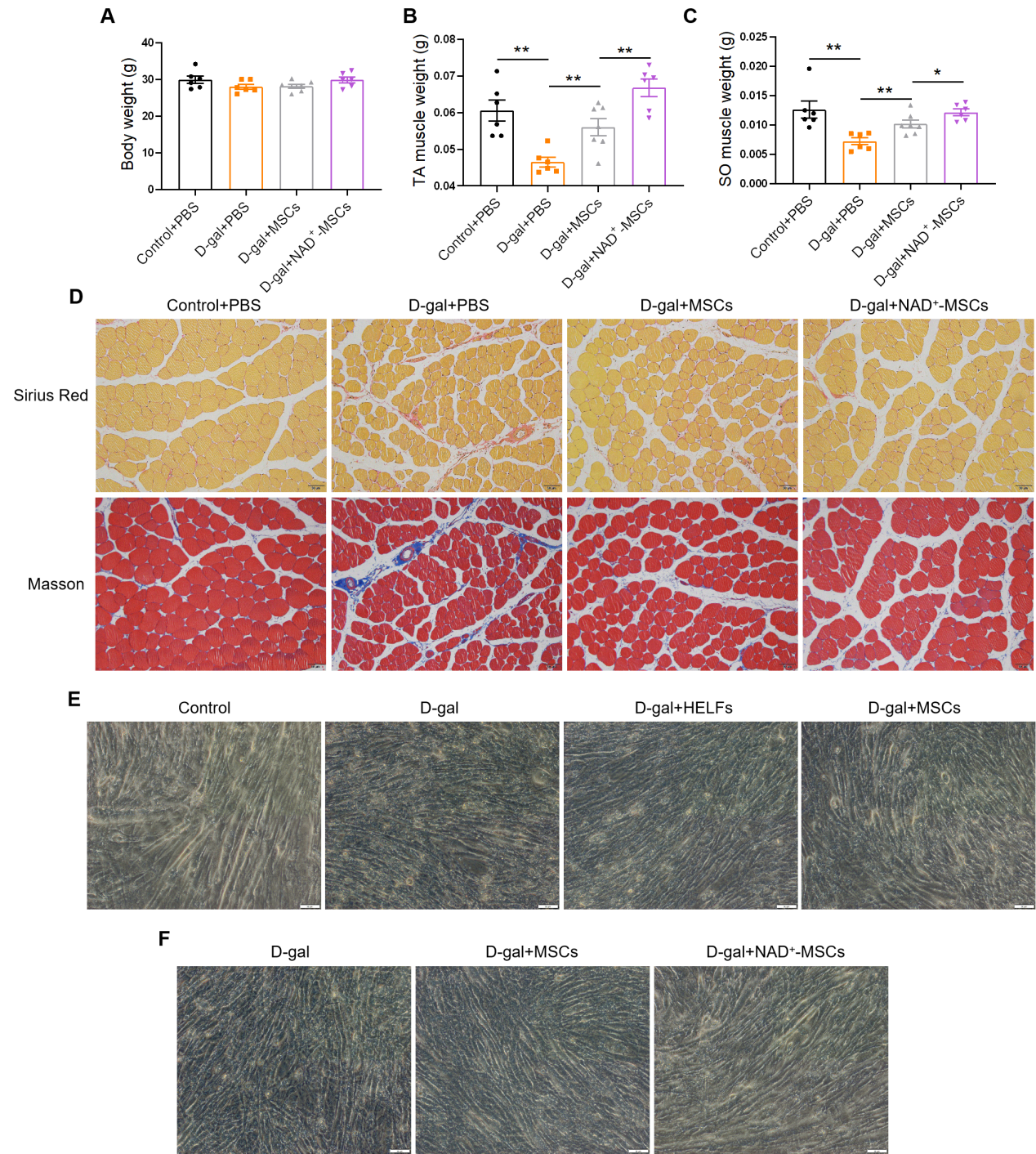
**Supplementary Figure 2. NAD^+^ pretreatment enhances the improvement effect of mesenchymal stromal cells (MSCs) on D-gal induced muscle atrophy**.(A) Body weight (n = 6–7 mice). (B) Tibialis anterior (TA) muscle weight (n = 6–7 mice). (C) Soleus (SO) muscle weight (n = 6–7 mice). (D) Sirius Red and Masson staining of TA muscles (Scale bar, 50 μm). (E) Representative images of C2C12 myotubes treated with D-gal and MSCs (Scale bar, 50 μm). (F) Representative images of C2C12 myotubes treated with MSCs and NAD^+^-MSCs (Scale bar, 50 μm). Data are presented as mean ± SEM. (**P* < 0.05, ***P* < 0.01)


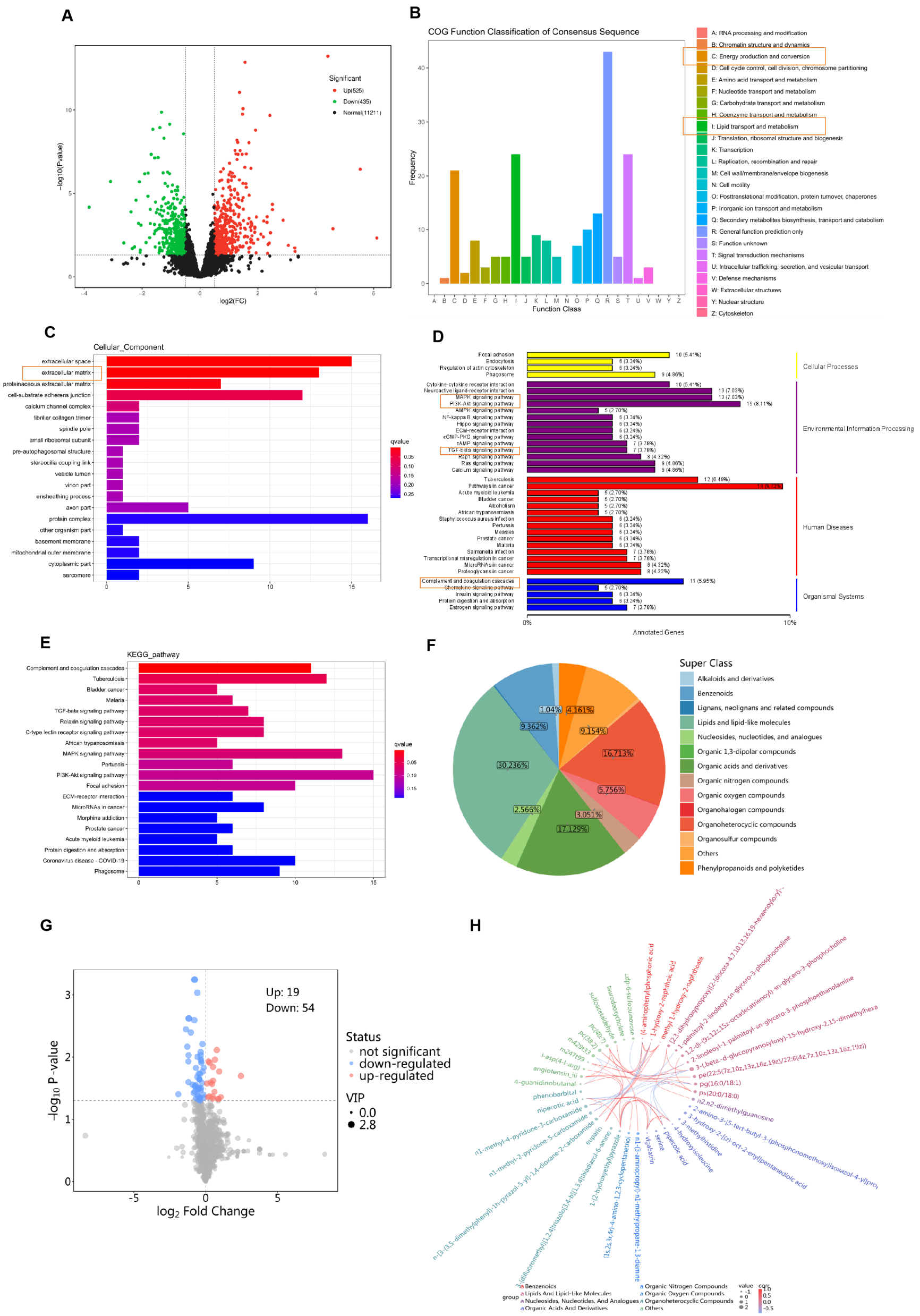


**Supplementary Figure 3. RNA-seq and metabolomics analysis of quadriceps (QUAD) muscle from D-gal mice with PBS or MSCs treatment.** (A) The volcano map of differentially expressed genes identified by RNA-seq analysis of quadriceps (QUAD) muscle from D-gal mice with PBS or MSCs treatment (n = 4 mice). (B) COG function classification of consensus sequence. (C) GO analysis of the down-regulated DEGs in cellular component. (D,E) KEGG analysis of the down-regulated DEGs. (F) Super class of metabolites identified by untargeted metabolomics of quadricep (QUAD) muscle from D-gal mice with PBS or MSCs treatment (n = 6 mice). (G) The volcano map of differentially expressed metabolites. (H) The chord plot of differentially expressed metabolites.


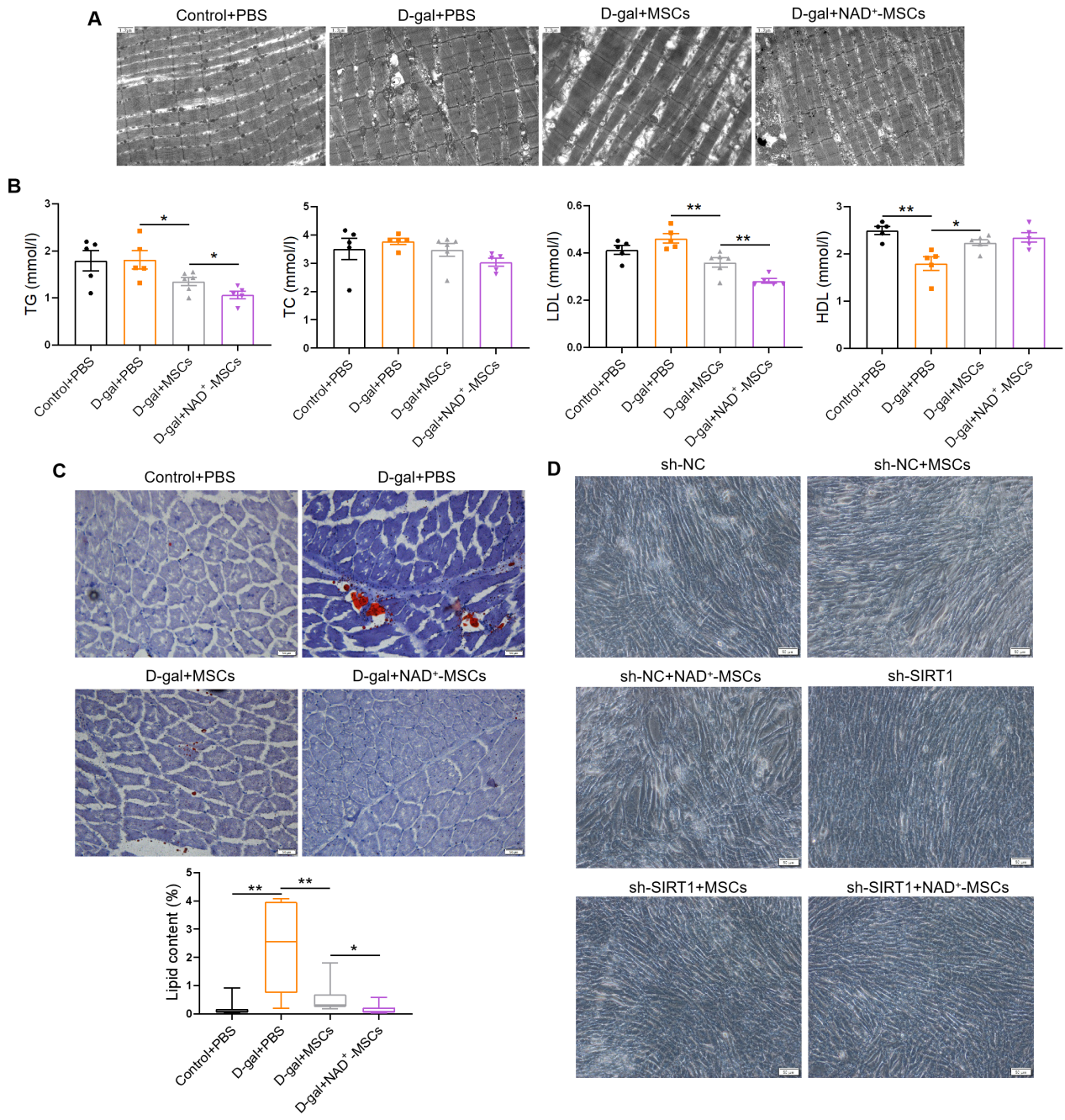
**Supplementary Figure 4. Mesenchymal stromal cells (MSCs)/NAD^+^-MSCs rescue muscle atrophy-associated impairment of mitochondrial function and lipid deposition through SIRT1**. (A) Transmission electron microscopy (TEM) images of intermyofibrillar (IMF) mitochondria in tibialis anterior (TA) muscles (Scale bar, 1.2 μm). (B) Plasma triglyceride (TG), total cholesterol (TC), low-density lipoprotein (LDL) and high**-**density lipoprotein (HDL) levels of mice (n = 5–6 mice). (C) Oil red O staining of QUAD muscles (Scale bar, 50 μm) and quantification of lipid content (n = 3 mice). (D) Representative images of C2C12 myotubes transfected with *SIRT1* shRNA and treated with MSCs/NAD^+^-MSCs (Scale bar, 50 μm). Data are presented as mean ± SEM. (**P* < 0.05, ***P* < 0.01)

**
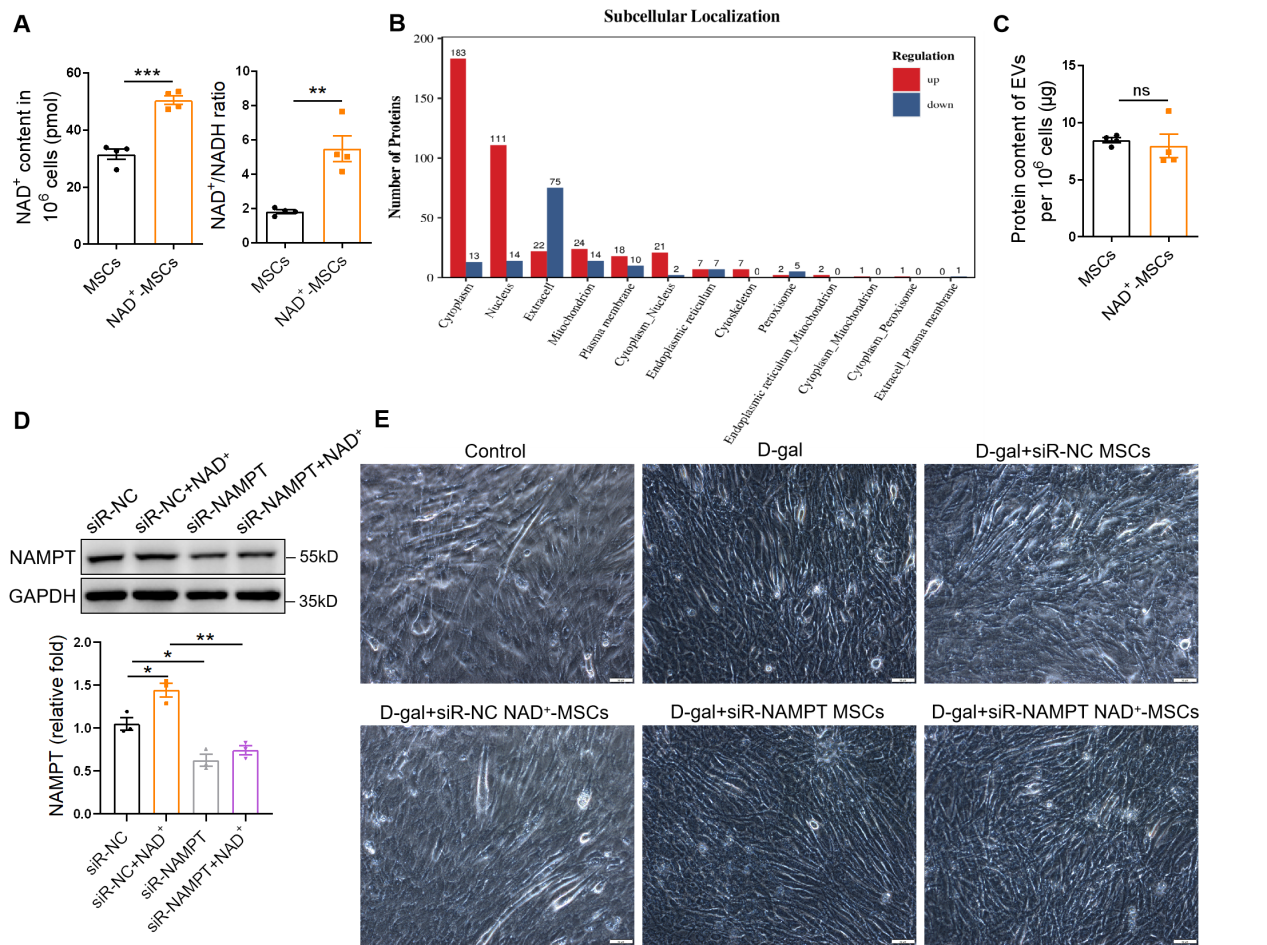
**

**Supplementary Figure 5. NAD^+^ enhances the therapeutic effect of mesenchymal stromal cells (MSCs) on muscle atrophy by promoting NAMPT secretion.** (A) Intracellular NAD^+^ content and NAD^+^/NADH ratio of MSCs with or without NAD^+^ treatment (n = 3). (B) Subcellular localization of protein component identified by supernatant proteomics analysis of MSCs with or without NAD^+^ treatment (n = 3). (C) The total protein content of EVs derived from MSCs with or without NAD^+^ treatment (n = 4). (D) Western blot analysis of NAMPT in MSCs transfected with NAMPT siRNA and treated with NAD^+^ (n=3). Quantification of bands was performed using ImageJ software. (E) Representative images of C2C12 myotubes treated with D-gal and siR-NAMPT MSCs/NAD^+^-MSCs (Scale bar, 50 μm). Data are presented as mean ± SEM. (**P* < 0.05, ***P* < 0.01, ****P* < 0.001)
