# Supplementary material for: NAD+ Enhanced Mesenchymal Stromal Cells Effect on Muscle Atrophy by Improving SIRT1‐Mediated Mitochondrial Function via NAMPT
Source: J Cachexia Sarcopenia Muscle. 2025 Dec 12;16(6):e70142. doi: 10.1002/jcsm.70142 (PMC12699140; doi:10.1002/jcsm.70142)
Supplement: Supplementary file 2 — Data S1: Supplementary References. [file JCSM-16-e70142-s001.docx]

**Supplementary references:**

[S1][Lim](https://pubmed.ncbi.nlm.nih.gov/?term=Lim+P&cauthor_id=39835720) P, [Woo](https://pubmed.ncbi.nlm.nih.gov/?term=Woo+SW&cauthor_id=39835720) SW, [Han](https://pubmed.ncbi.nlm.nih.gov/?term=Han+J&cauthor_id=39835720) J, [Lee](https://pubmed.ncbi.nlm.nih.gov/?term=Lee+YL&cauthor_id=39835720) YL, [Shim](https://pubmed.ncbi.nlm.nih.gov/?term=Shim+JH&cauthor_id=39835720) JH, [Kim](https://pubmed.ncbi.nlm.nih.gov/?term=Kim+HS&cauthor_id=39835720) HS. Danshensu sodium salt alleviates muscle atrophy via CaMKII-PGC1α-FoxO3a signaling pathway in D-galactose-induced models. FASEB J. 2025 Jan 31;39(2):e70280. DOI: 10.1096/fj.202402158R.

[S2]Jiang X, Ji S, Yuan F, Li T, Cui S, Wang W, et al. Pyruvate dehydrogenase B regulates myogenic differentiation via the FoxP1-Arih2 axis. J Cachexia Sarcopenia Muscle. 2023 Feb;14(1):606-621. DOI: 10.1002/jcsm.13166.

[S3]Wang HH, Zhang Y, Qu TQ, Sang XQ, Li YX, Ren FZ, et al. Nobiletin Improves D-Galactose-Induced Aging Mice Skeletal Muscle Atrophy by Regulating Protein Homeostasis. Nutrients. 2023 Apr 7;15(8):1801. DOI: 10.3390/nu15081801.

[S4]Takegaki J, Sase K, Kono Y, Nakano D, Fujita T, Konishi S, et al. Intramuscular injection of mesenchymal stem cells activates anabolic and catabolic systems in mouse skeletal muscle. Scientific reports. 2021, 11(1): 21224. [DOI: 10.1038/s41598-021-00627-6.](https://doi.org/10.1038/s41598-021-00627-6)

[S5]Wang H, Sun Y, Pi C, Yu X, Gao X, Zhang C, et al. Nicotinamide Mononucleotide Supplementation Improves Mitochondrial Dysfunction and Rescues Cellular Senescence by NAD/Sirt3 Pathway in Mesenchymal Stem Cells. Int J Mol Sci. 2022, 23(23):14739. [DOI: 10.3390/ijms232314739.](https://doi.org/10.3390/ijms232314739)

[S6]Chong MC, Silva A, James PF, Wu SSX, Howitt J. [Exercise increases the release of NAMPT in extracellular vesicles and alters NAD](https://pubmed.ncbi.nlm.nih.gov/35661560/)^[+](https://pubmed.ncbi.nlm.nih.gov/35661560/)^[activity in recipient cells.](https://pubmed.ncbi.nlm.nih.gov/35661560/) Aging Cell. 2022, 21(7):e13647. DOI: 10.1111/acel.13647.

[S7]Short KR, Bigelow ML, Kahl J, Singh R, Coenen-Schimke J, Raghavakaimal S, et al. Decline in skeletal muscle mitochondrial function with aging in humans. Proc Natl Acad Sci U S A. 2005, 102:5618-5623. [DOI: 10.1073/pnas.0501559102.](https://doi.org/10.1073/pnas.0501559102)
