# Supplementary material for: NAD+ Enhanced Mesenchymal Stromal Cells Effect on Muscle Atrophy by Improving SIRT1‐Mediated Mitochondrial Function via NAMPT
Source: J Cachexia Sarcopenia Muscle. 2025 Dec 12;16(6):e70142. doi: 10.1002/jcsm.70142 (PMC12699140; doi:10.1002/jcsm.70142)

Figure 1

Figure 1D

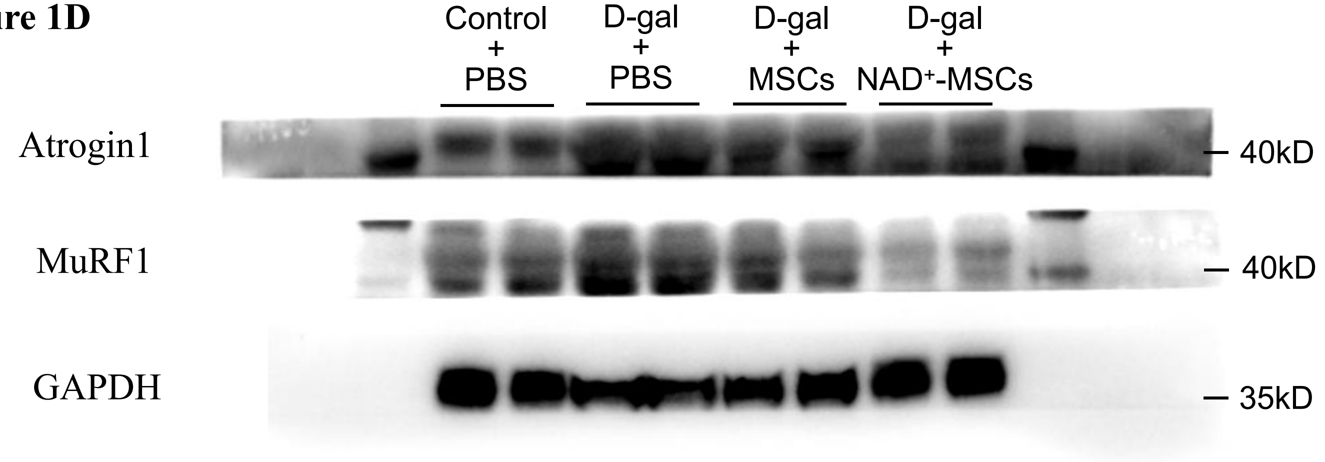

Figure 1E

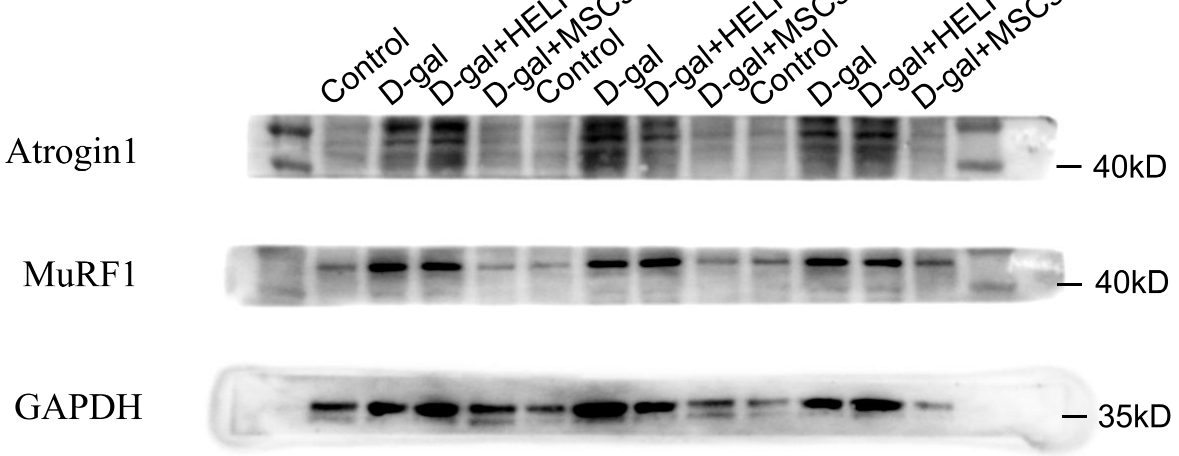

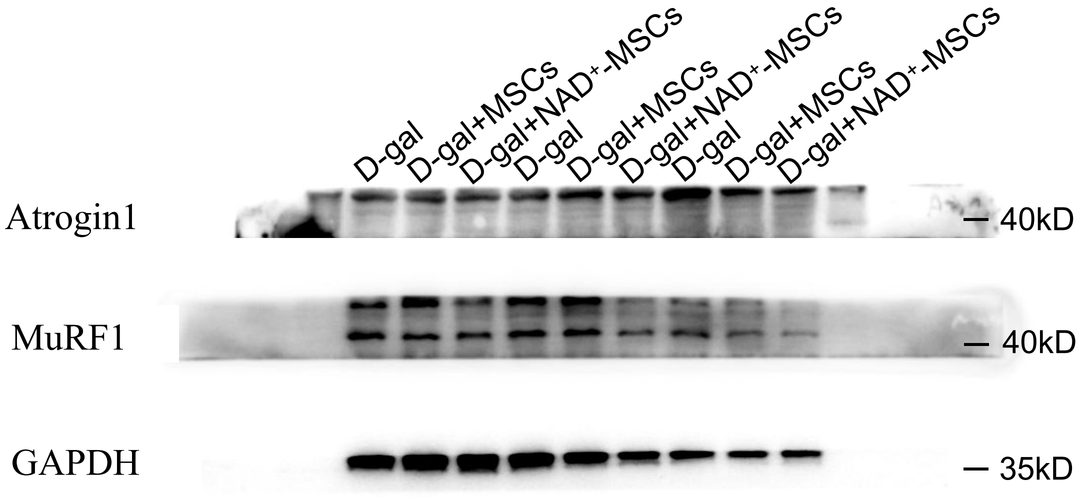

Figure 2

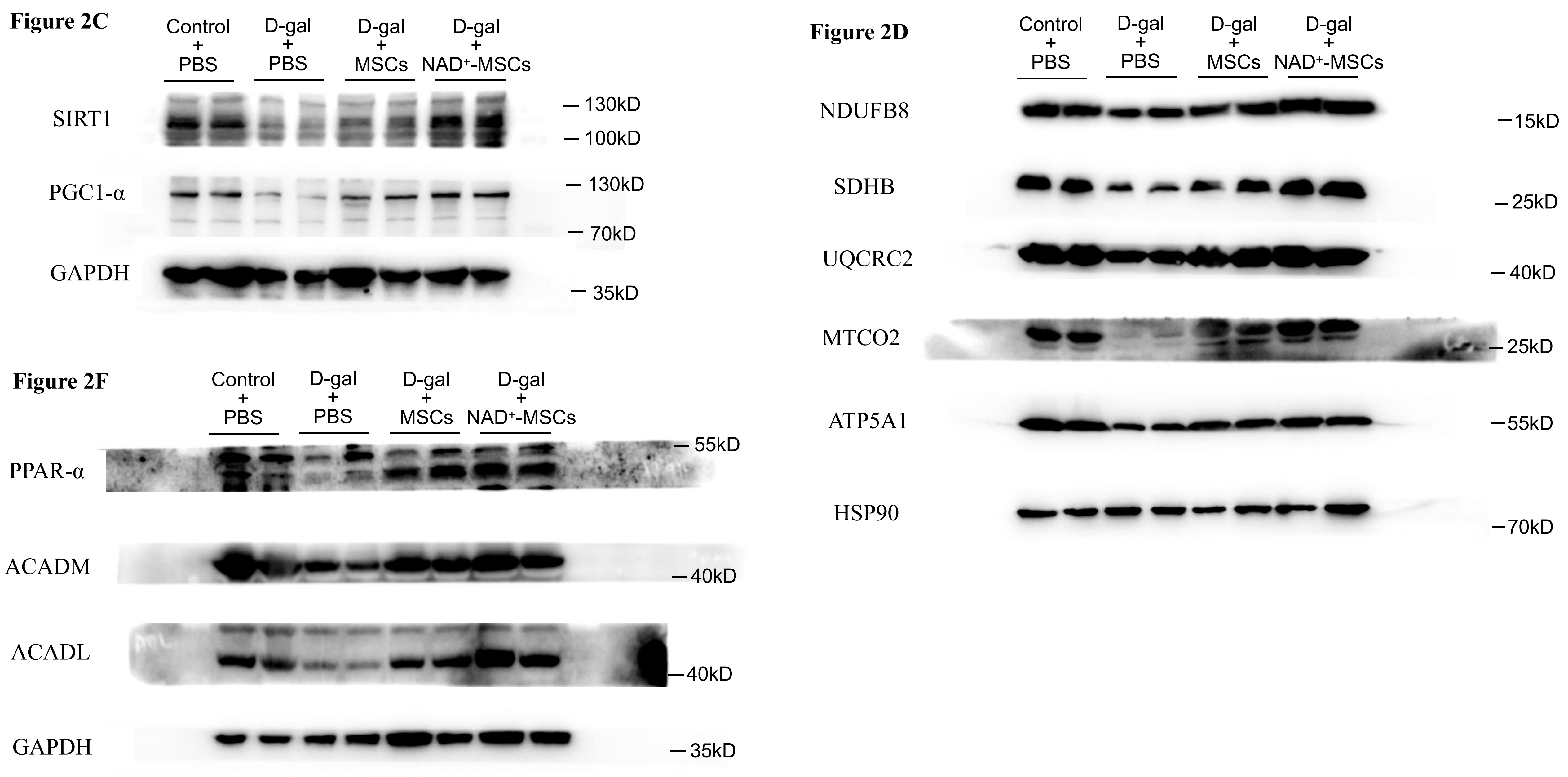
Figure 2C
Control + PBS
D-gal + PBS
D-gal + MSCs
D-gal + NAD⁺-MSCs
SIRT1
PGC1-α
GAPDH
130kD
100kD
130kD
70kD
35kD
Figure 2D
Control + PBS
D-gal + PBS
D-gal + MSCs
D-gal + NAD⁺-MSCs
NDUFB8
SDHB
UQCRC2
MTCO2
ATP5A1
HSP90
15kD
25kD
40kD
25kD
55kD
70kD
Figure 2F
Control + PBS
D-gal + PBS
D-gal + MSCs
D-gal + NAD⁺-MSCs
PPAR-α
ACADM
ACADL
GAPDH
55kD
40kD
40kD
35kD

Figure 3

Figure 3A

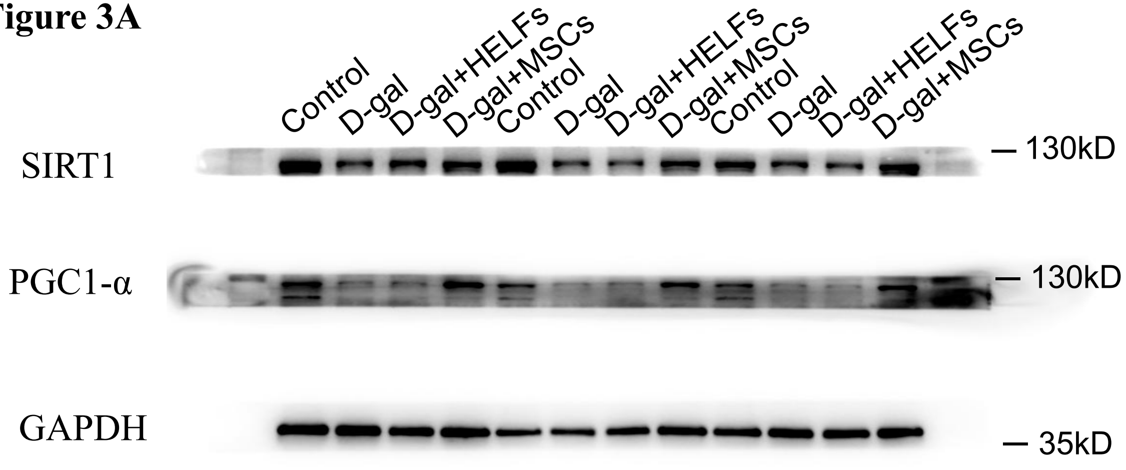

Figure 3B

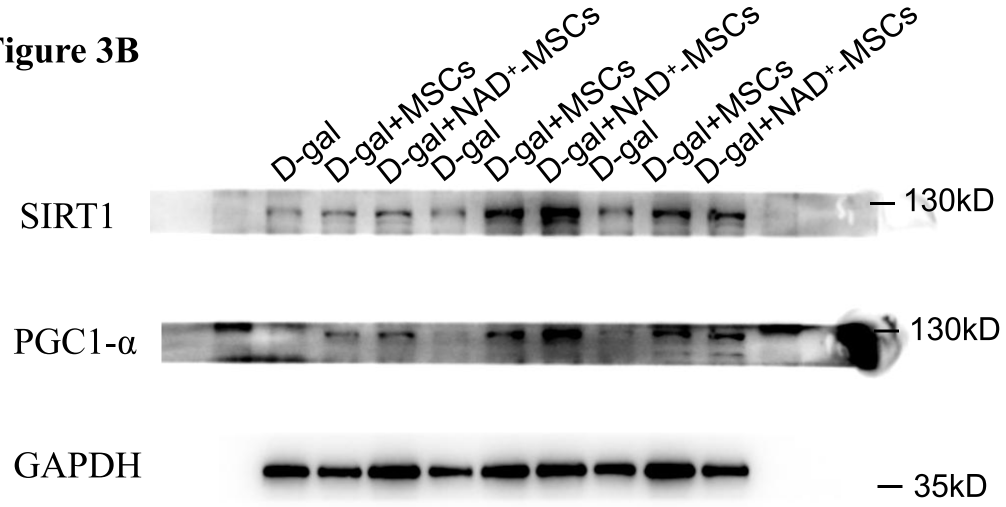

Figure 3C

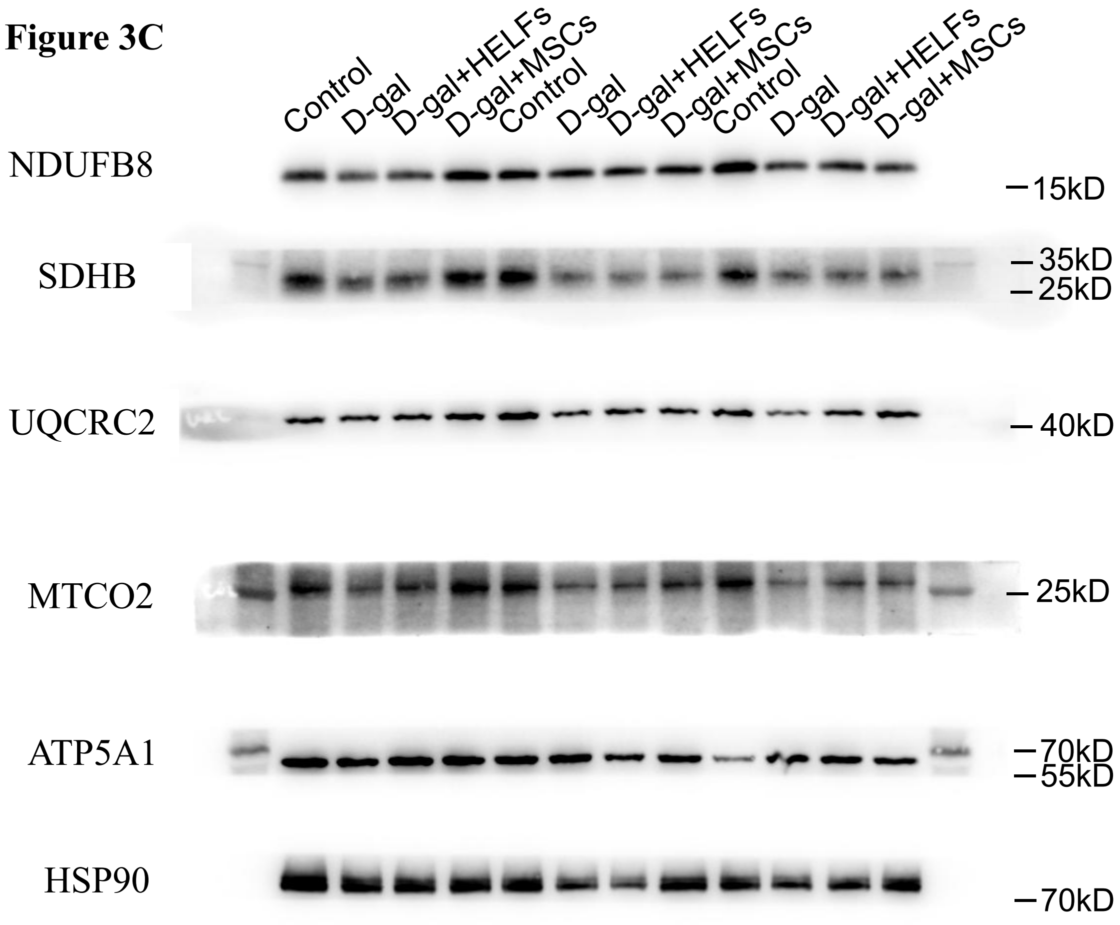

Figure 3

Figure 3D

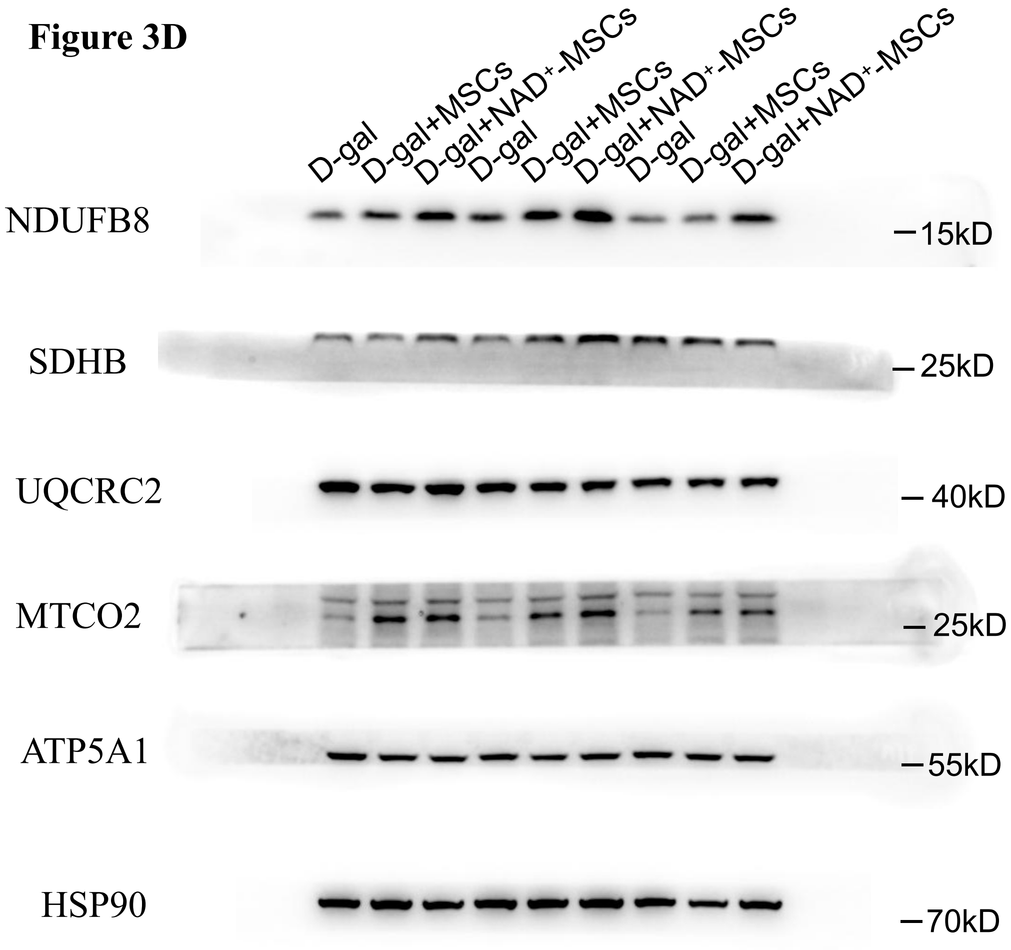

Figure 3E

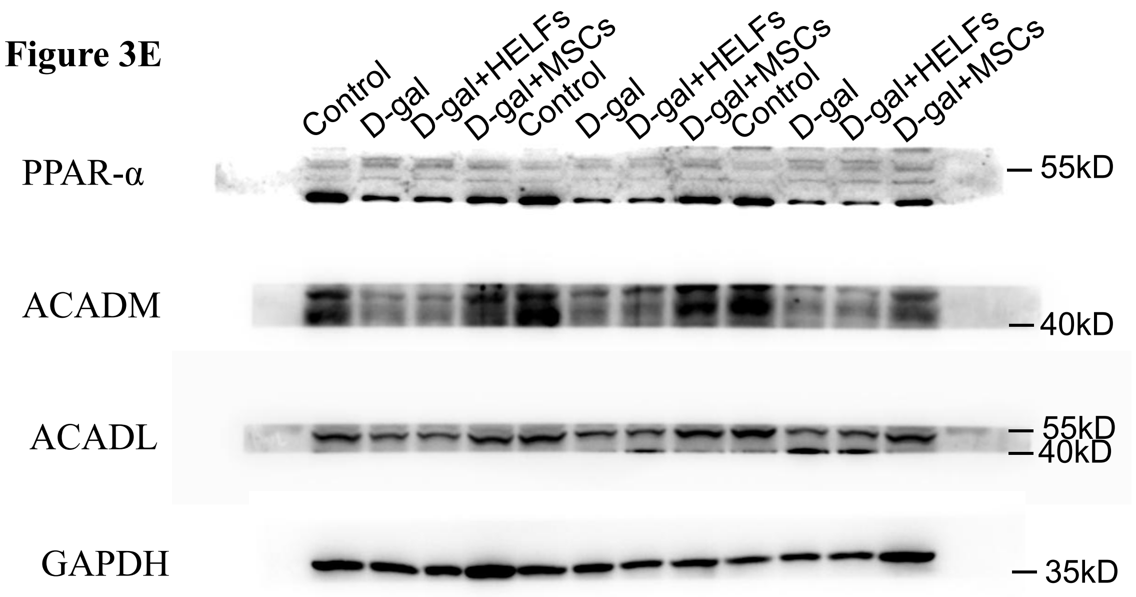

Figure 3F

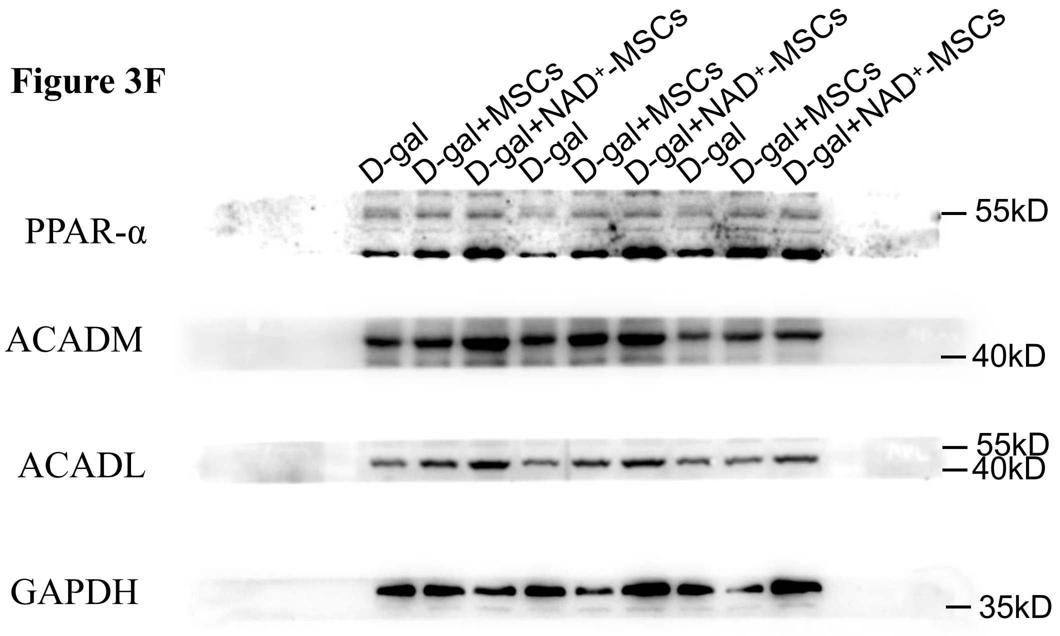

Figure 4

Figure 4A

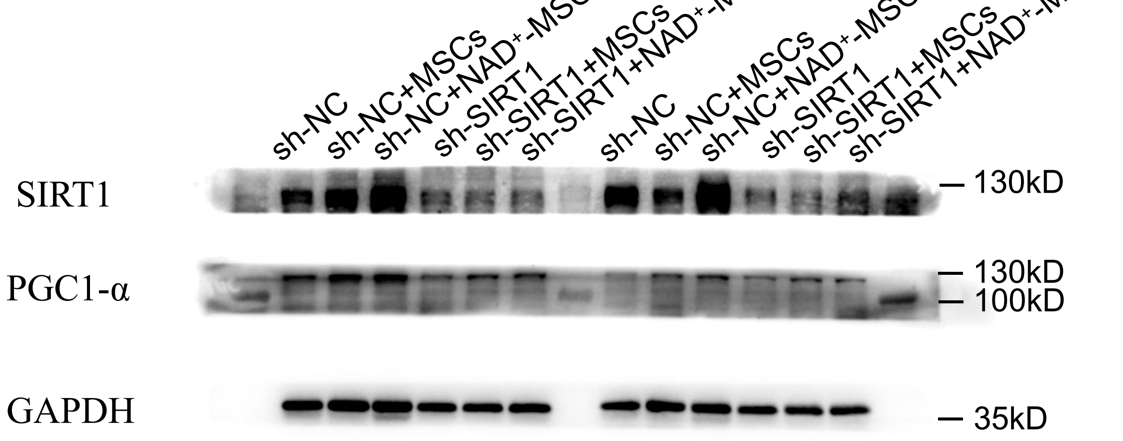

Figure 4B

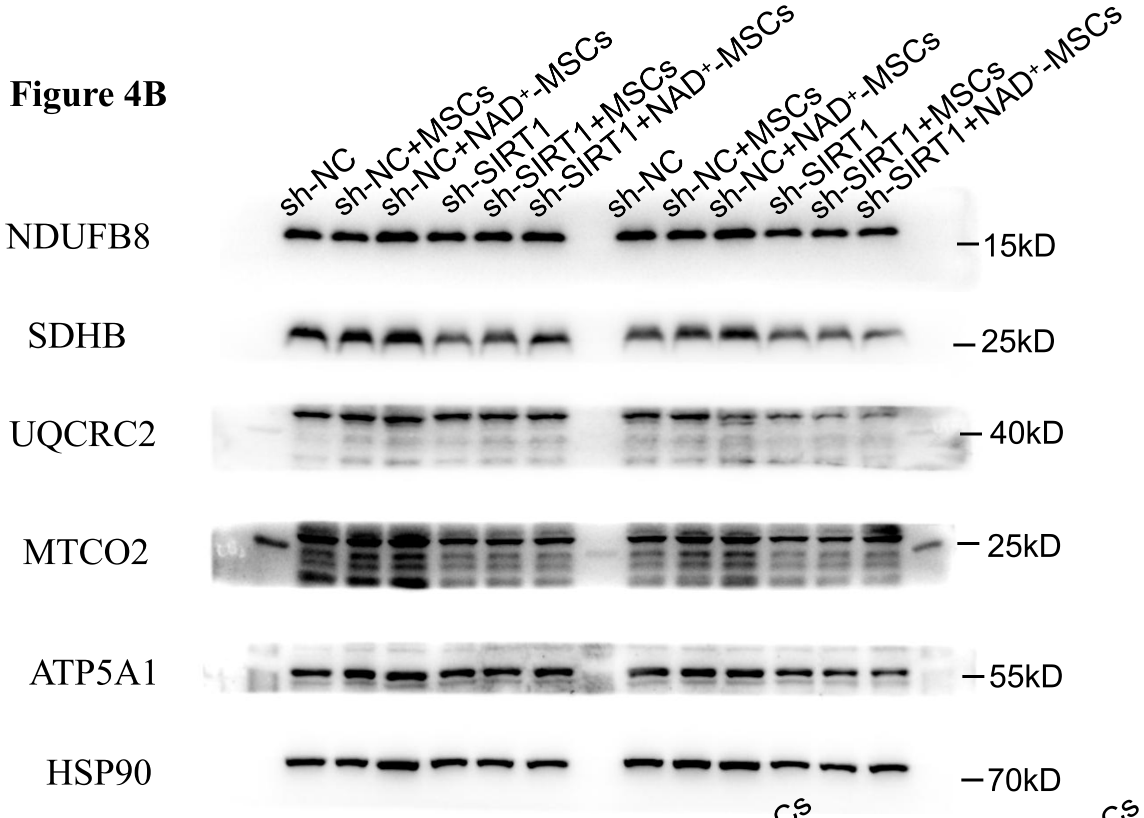

Figure 4D

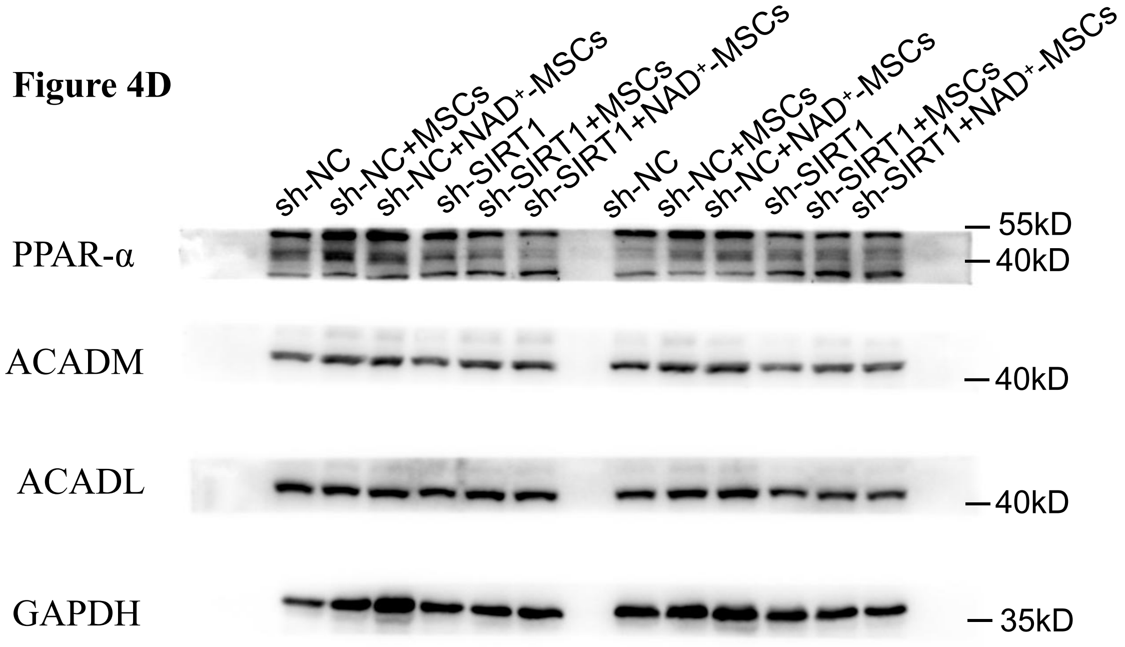

Figure 4E

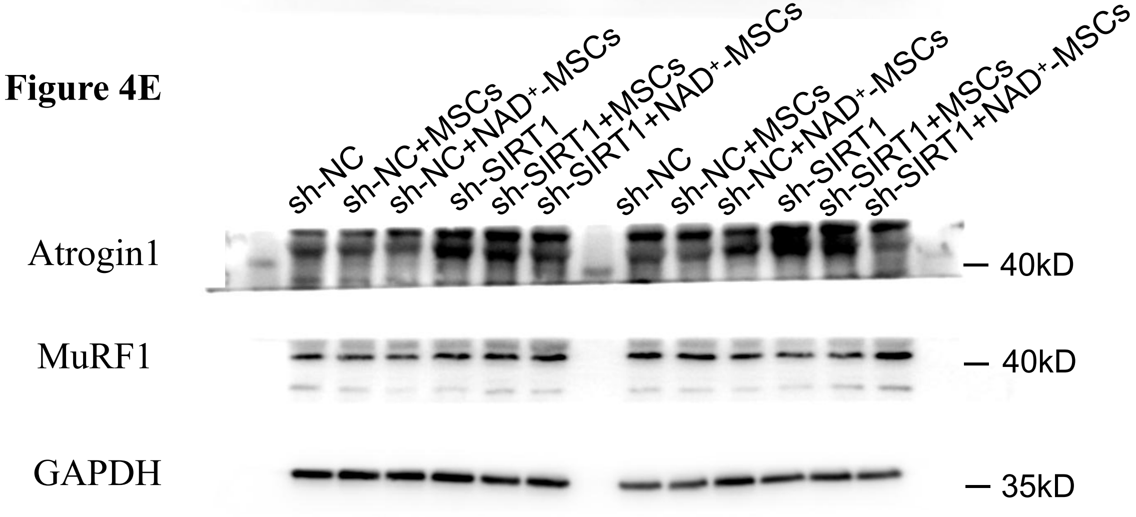

Figure 5

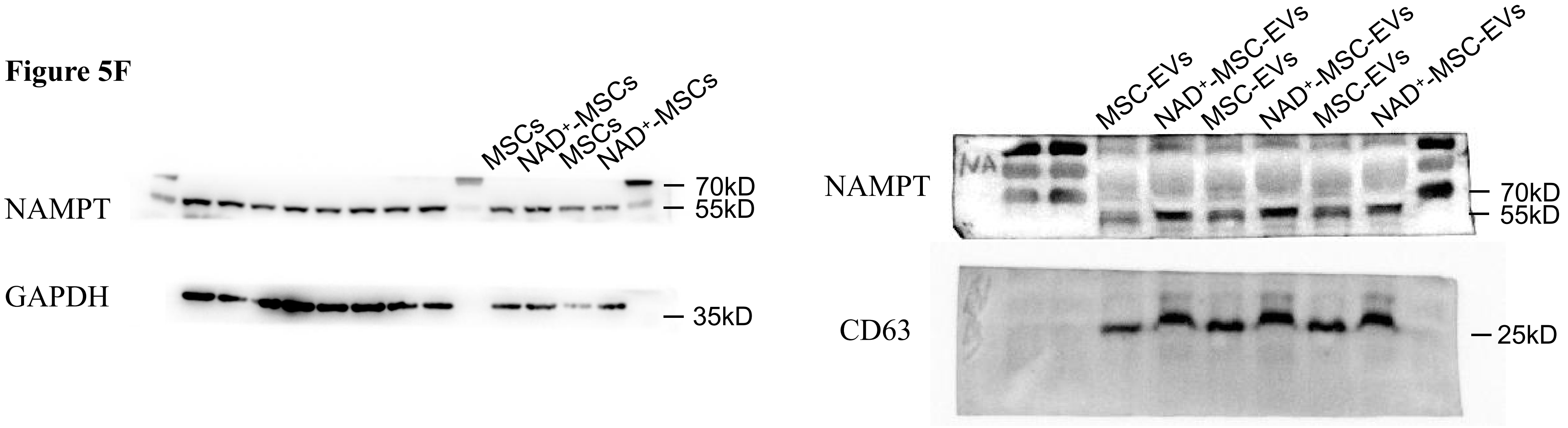

Figure 5F
MSCs
NAD⁺-MSCs
MSCs
NAD⁺-MSCs
NAMPT
70kD
55kD
GAPDH
35kD
MSC-EVs
NAD⁺-MSC-EVs
MSC-EVs
NAD⁺-MSC-EVs
MSC-EVs
NAD⁺-MSC-EVs
NAMPT
70kD
55kD
CD63
25kD

Figure 6

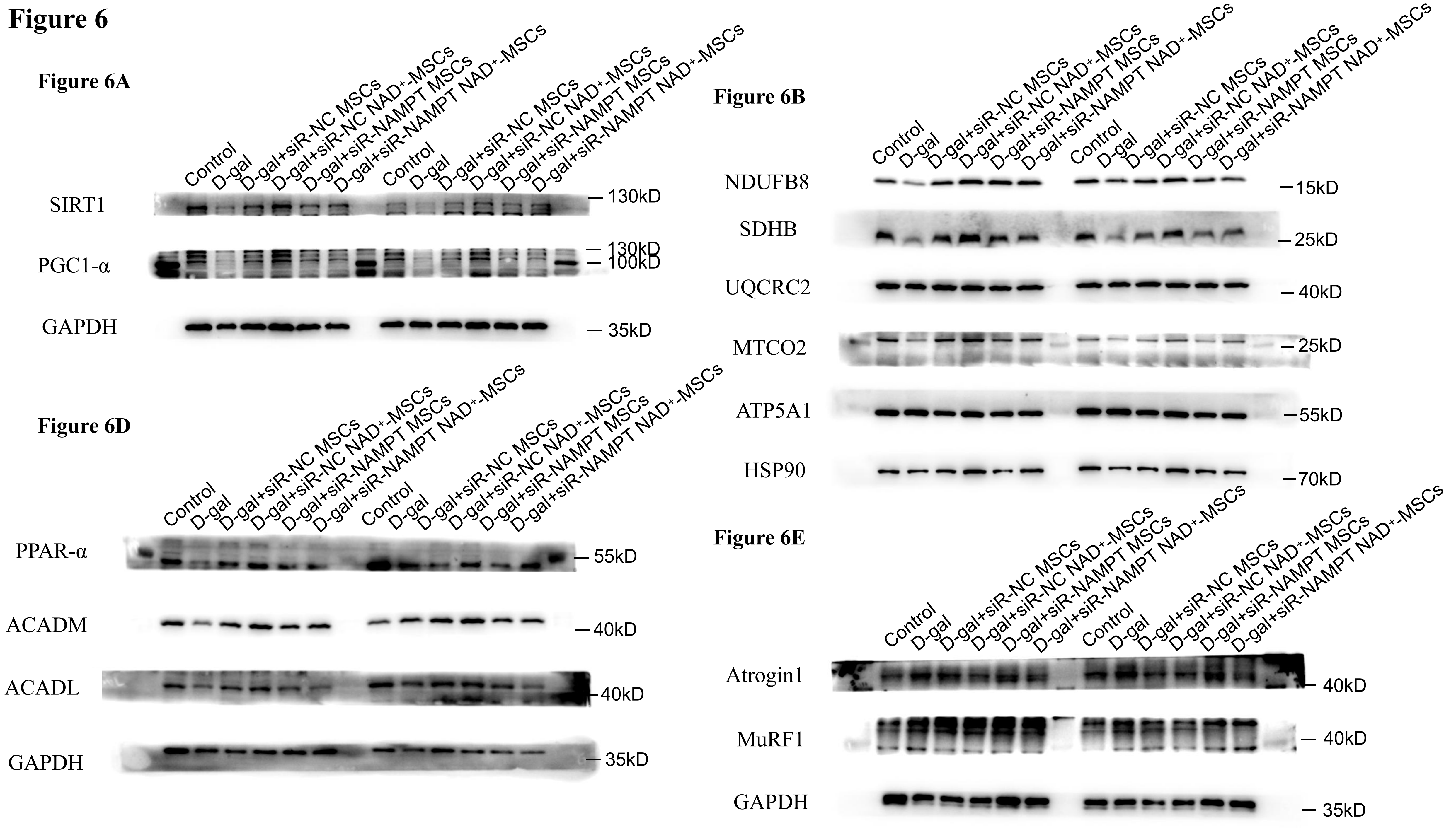
Figure 6A
Control
D-gal
D-gal+siR-NC MSCs
D-gal+siR-NC NAD⁺-MSCs
D-gal+siR-NAMPT MSCs
D-gal+siR-NAMPT NAD⁺-MSCs
SIRT1
130kD
PGC1-α
130kD
100kD
GAPDH
35kD
Figure 6B
NDUFB8
15kD
SDHB
25kD
UQCRC2
40kD
MTCO2
25kD
ATP5A1
55kD
HSP90
70kD
Figure 6D
PPAR-α
55kD
ACADM
40kD
ACADL
40kD
GAPDH
35kD
Figure 6E
Atrogin1
40kD
MuRF1
40kD
GAPDH
35kD

Figure S5

Figure S5C

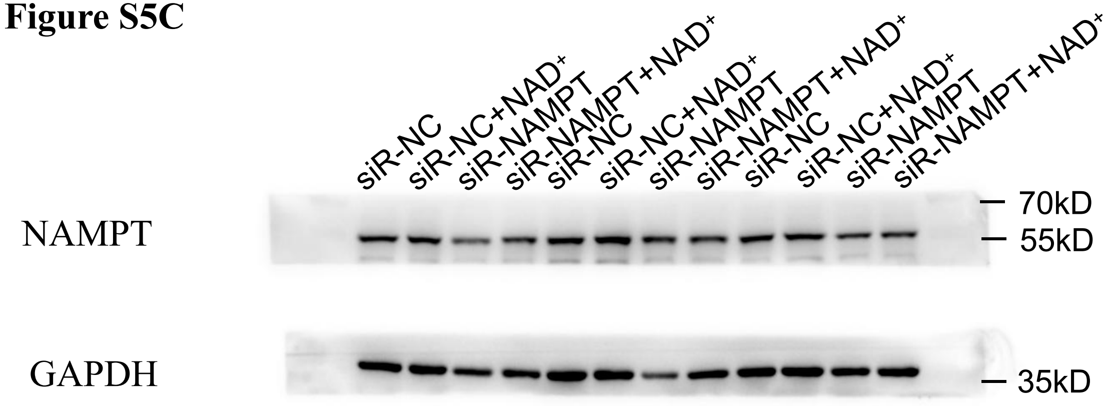

Supplement: Supplementary file 3 — Data S2: Supplementary Information. [file JCSM-16-e70142-s002.pdf]
